# A DNA methylation state transition model reveals the programmed epigenetic heterogeneity in human pre-implantation embryos

**DOI:** 10.1186/s13059-020-02189-8

**Published:** 2020-11-16

**Authors:** Chengchen Zhao, Naiqian Zhang, Yalin Zhang, Nuermaimaiti Tuersunjiang, Shaorong Gao, Wenqiang Liu, Yong Zhang

**Affiliations:** 1 Institute for Regenerative Medicine, Shanghai East Hospital, Shanghai Key Laboratory of Signaling and Disease Research, Frontier Science Center for Stem Cell Research, School of Life Science and Technology, Tongji University, Shanghai, 200092 China; 2 School of Mathematics and Statistics, Shandong University at Weihai, Weihai, 264209 China; 3 Clinical and Translational Research Center of Shanghai First Maternity and Infant Hospital, Tongji University, Shanghai, 200092 China

**Keywords:** DNA methylation, Heterogeneity, First cell fate determination

## Abstract

**Background:**

During mammalian early embryogenesis, expression and epigenetic heterogeneity emerge before the first cell fate determination, but the programs causing such determinate heterogeneity are largely unexplored.

**Results:**

Here, we present MethylTransition, a novel DNA methylation state transition model, for characterizing methylation changes during one or a few cell cycles at single-cell resolution. MethylTransition involves the creation of a transition matrix comprising three parameters that represent the probabilities of DNA methylation-modifying activities in order to link the methylation states before and after a cell cycle. We apply MethylTransition to single-cell DNA methylome data from human pre-implantation embryogenesis and elucidate that the DNA methylation heterogeneity that emerges at promoters during this process is largely an intrinsic output of a program with unique probabilities of DNA methylation-modifying activities. Moreover, we experimentally validate the effect of the initial DNA methylation on expression heterogeneity in pre-implantation mouse embryos.

**Conclusions:**

Our study reveals the programmed DNA methylation heterogeneity during human pre-implantation embryogenesis through a novel mathematical model and provides valuable clues for identifying the driving factors of the first cell fate determination during this process.

**Supplementary information:**

**Supplementary information** accompanies this paper at 10.1186/s13059-020-02189-8.

## Background

During mammalian pre-implantation embryogenesis, gene transcription regulation undergoes dramatic reprogramming [1–3], and expression heterogeneity emerges among cells within the same embryo before the first cell fate determination, i.e., the separation between the inner cell mass (ICM) and trophectoderm (TE) [4, 5]. A few reports have suggested that such heterogeneity could affect the first cell fate determination. For example, expression heterogeneity of *Carm1* in the 4-cell stage mouse embryo has been reported to cause later imbalanced expression of *Sox21*, which is critical in the determination of cell differentiation into ICM or TE cells [6, 7]. Two recent studies have indicated that the cause of *Carm1* expression heterogeneity can be traced back to the 2-cell stage and is associated with the expression heterogeneity of two long non-coding RNAs, *LincGET* [8] and *Neat1* [9]. Considering the robustness of early embryogenesis, such expression heterogeneity has been suggested to be the determinate result of a programmed process [10, 11], i.e., a set of cellular instructions. However, to the best of our knowledge, the suggested program causing such heterogeneity is largely unexplored. In addition to gene expression levels, DNA methylation levels have been reported to be heterogeneous among cells in pre-implantation embryos [12], likely as a result of a programmed process. As DNA methylation plays important roles in gene transcriptional regulation, the expression heterogeneity that emerges during early embryogenesis could at least partially be explained by DNA methylation. In contrast to gene expression, DNA methylation exists in a binary state for each CpG dyad, and the transition of its state during each cell cycle can be described quantitatively [13]. In addition, DNA methylation can be measured at a single CpG resolution in single cells [12, 14–16]. Therefore, it is promising to elucidate the set of instructions causing such epigenetic heterogeneity during early embryogenesis by building a mathematical model.

Several mathematical models have been built to quantitatively describe the DNA methylation state transition across cell cycles [17–24]. Two models built in the early 1990s specified that the DNA methylation state in a CpG dyad can be one of three states, unmethylated, hemi-methylated, and methylated, and that the type of DNA methylation state transition can be either de novo methylation or methylation maintenance [17, 18]. Genereux et al. and Sontag et al. improved the models by considering de novo methylation on different DNA strands separately [19, 20]. Fu et al. and Arand et al. considered the discriminates among the DNA methyltransferases (DNMTs) based on hairpin bisulfite sequencing (hairpin-BS-seq) data [21, 22]. McGovern et al. and von Meyenn et al. further incorporated 5-hydroxymethylcytosine (5hmC) into models to reflect the contribution of active demethylation to the DNA methylation state transition [23, 24]. Recently, Busto-Moner et al. enabled genome-wide quantification of subcell cycle kinetics of methylation maintenance using replication-associated bisulfite sequencing (Repli-BS-seq) data [25]. Researchers have proposed two types of methods to estimate the parameters of the models. One type of method relies on the acquisition of a DNA methylation equilibrium state. For example, Genereux et al. applied a maximum likelihood to estimate the parameters when the equilibrium state was reached [19], and Sontag et al. used a Markov chain model with a defined steady state [20]. The other type relies on the availability of specific genomics data. For example, Arand et al. calculated the efficiencies of DNMTs, i.e., the parameters of the transition model, based on hairpin-BS-seq data upon knockout (KO) of each DNMT [22]. von Meyenn et al. applied partial differential equations to estimate the parameters by using reduced representation bisulfite sequencing (RRBS) data, hairpin-BS-seq data, and TET-assisted bisulfite sequencing (TAB-seq) data [24]. Busto-Moner et al. used maximum likelihood estimation (MLE) to estimate the parameters quantifying the remethylation kinetics of nascent DNA post-replication relied on Repli-BS-seq data [25].

Although several mathematical models are available to describe the DNA methylation state transition, none of them can be applied to study DNA methylation heterogeneity during mammalian early embryogenesis using single-cell DNA methylome data, mainly for three reasons. First, the DNA methylation level changes dramatically across each cell cycle during early embryogenesis, demonstrating that the DNA methylation level is far from an equilibrium state. Therefore, models that incorporate parameter estimation methods based on an equilibrium state are not suitable. Second, active removal of DNA methylation is prevalent during mammalian early embryogenesis [1–3]; thus, models that do not consider active demethylation cannot be applied to this process. Third, due to technical limitations, technologies for profiling 5-methylcytosine (5mC) and 5hmC states together within the same cell are still not available, so models requiring both 5mC and 5hmC information are not suitable. Overall, a new DNA methylation state transition model is needed to study DNA methylation heterogeneity during mammalian early embryogenesis.

In this study, we developed a DNA methylation state transition model, MethylTransition, for characterizing methylation changes during one or a few cell cycles at single-cell resolution. MethylTransition introduces a methylation state ratio vector with 5 discrete states to describe the overall pattern of DNA methylation states for a given cell. To link the two methylation state ratio vectors before and after a cell cycle, MethylTransition uses a transition matrix comprising 3 parameters separately representing the probabilities of DNA methylation maintenance, active demethylation, and de novo methylation. The model estimates the parameters via a matrix approximation strategy with the Newton-Raphson method. Unlike previous models, MethylTransition does not need an equilibrium state or a 5hmC profile. Therefore, it is suitable for the elucidation of DNA methylation heterogeneity during mammalian early embryogenesis based on single-cell bisulfite sequencing (scBS-seq) data. We applied MethylTransition to scBS-seq data during human pre-implantation embryogenesis, and we found that the DNA methylation heterogeneity that emerges is largely determined by the initial DNA methylation state at the zygote stage (i.e., the 1-cell stage) and by a set of instructions represented by the model parameters. We further discussed the potential regulatory effects of DNA methylation heterogeneity in early embryos. The source code for MethylTransition can be found at https://github.com/TongjiZhanglab/MethylTransition [26].

## Results

### Computational framework of MethylTransition

**Fig. 1:**
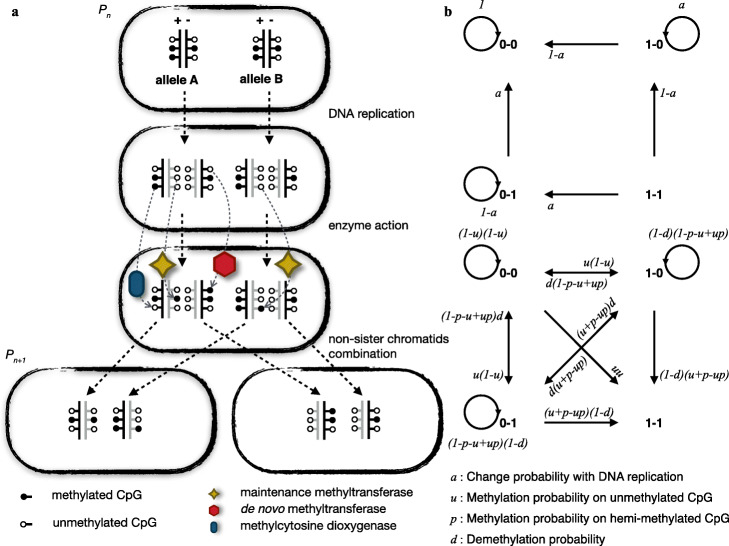
Computational framework of MethylTransition. **a** Schematic overview of DNA methylation changes during mitosis. MethylTransition relies on the assumption that changes in the DNA methylation state at a CpG site across a single cell cycle occur in three steps: passive demethylation during DNA replication, active DNA methylation by DNA methylation-modifying enzymes, and DNA methylation state combination during the combination of non-sister chromatids. The open circle represents a CpG dinucleotides with an unmethylated C, and the solid circle represents a CpG dinucleotides with a methylated C. In the first step, DNA replication, the newly synthetized strand (gray) is unmethylated. In the second step, enzyme action, the DNA methylation state in a CpG dyad can be changed by different enzymes. In the third step, non-sister chromatid combination, the observed DNA methylation state of a CpG site in a single cell is the combination of the DNA methylation states from both homologous chromosomes for diploid organisms. **b** Transition probabilities among DNA methylation states during DNA replication and enzyme action. We used *1* as a symbol to represent a CpG dinucleotides with a methylated C and *0* to represent a CpG dinucleotides with an unmethylated C. The DNA methylation state for a CpG dyad can be unmethylated (*0-0*), hemi-methylated (*0-1* or *1-0*), or fully methylated (*1-1*). The upper panel shows the transition probabilities among states during DNA replication, where *a* represents the probability of parental DNA being present in one of two daughter strands and is 0.5 for the symmetric division. The lower panel shows the transition probabilities among states during enzyme action, where *u* is the probability of de novo methylation on an unmethylated CpG dyad, *p* is the probability of methylation maintenance on a hemi-methylated CpG dyad, and *d* is the probability of active demethylation on a methylated or hemi-methylated CpG dyad

To quantitatively characterize the DNA methylation state transition across a single cell cycle based on scBS-seq data, we developed MethylTransition, a probabilistic model with robustly estimated parameters. MethylTransition relies on the assumption that changes in the DNA methylation state at a CpG site across a single cell cycle occur in three steps: passive demethylation during DNA replication, active DNA methylation alteration by DNA methylation-modifying enzymes, and DNA methylation state combination during the combination of non-sister chromatids (Fig. 1a). A CpG site contains two CpG dyads in a diploid cell, while a CpG dyad contains two complemented CpG dinucleotides (Additional file 1: Fig. S1a). To illustrate the state transition probability at each step, we used *1* as a symbol to represent methylated CpG dinucleotides and *0* to represent unmethylated CpG dinucleotides. Thus, the DNA methylation state for a CpG dyad can be one of the following types: unmethylated (*0-0*), hemi-methylated (*0-1* or *1-0*), or fully methylated (*1-1*). In the first step, i.e., DNA replication, MethylTransition assumes that the newly synthetized strand is unmethylated. The transition probabilities among DNA methylation states for a CpG dyad are displayed in the upper panel of Fig. 1b (see the “Methods” section for details), where *a* represents the probability of parental DNA in one of two daughter strands and is 0.5 for the symmetric division. In the second step, i.e., enzyme action, the DNA methylation state in a CpG dyad can be changed by different enzymes. According to the distinct roles of DNA methylation-modifying enzymes, MethylTransition denotes the probability of de novo methylation on an unmethylated CpG dyad as *u*, the probability of methylation maintenance on a hemi-methylated CpG dyad as *p*, and the probability of active demethylation on a methylated or hemi-methylated CpG dyad as *d*. The transition probabilities among DNA methylation states for a CpG dyad are shown in the lower panel of Fig. 1b (see the “Methods” for details). In the third step, i.e., non-sister chromatid combination, the observed DNA methylation state of a CpG site in a single cell is the combination of the DNA methylation states of both homologous chromosomes for diploid organisms. There are five possible methylation states at each CpG site: unmethylated (labeled S1), quarter-methylated (S2), half-methylated (S3), three-quarter-methylated (S4), and fully methylated (S5) (Additional file 1: Fig. S1a; see the “Methods” section for details). Then, MethylTransition generates a matrix to describe the transition probability among the five states in this step (see the “Methods” section for details). Overall, MethylTransition constructs a probability matrix (*T*_*A*_) with introduced parameters to quantitatively characterize the DNA methylation state transition across a single cell cycle.

Within the framework of MethylTransition, a transition frequency matrix could theoretically be calculated by measuring the DNA methylation state of a CpG site before and after a cell cycle, and then the parameters *u*, *d*, and *p* could be estimated. However, due to the high dropout rate of public scBS-seq data, the percentage of CpG sites both sequenced in two scBS-seq datasets is generally less than 20% (Additional file 1: Fig. S1b), and it is difficult to accurately assign a methylation state to a CpG site based on scBS-seq data. To overcome the above difficulties, we propose two assumptions. First, the DNA methylation states of CpG sites in the promoter of a gene can be represented by the average DNA methylation level in this promoter. This assumption is largely true for the mammalian genome [27], and the various DNA methylation levels in promoters are much smaller than those in other regions (Additional file 1: Fig. S1c). Second, the activity levels of DNA methylation-modifying enzymes in genomic regions with similar chromatin status are largely consistent. Based on these two assumptions, for genes with similar chromatin status at their promoters, each gene’s promoter can be assigned one of the five methylation states (S1 to S5) using scBS-seq data, and the methylation state ratio vector of the genes in each methylation state can be used to present the overall pattern of methylation states for those genes within a given cell (see the “Methods” section for details). Given two separate methylation state ratio vectors before and after a cell cycle, the transition frequency matrix *T*_*B*_ linking the two vectors can be calculated. Then, MethylTransition can estimate the parameters *u*, *d*, and *p* by minimizing the difference between the transition probability matrix *T*_*A*_ and the calculated transition frequency matrix *T*_*B*_, while a cost function is constructed and optimized by using the Newton-Raphson method [28] (see “Methods” section for details). In addition to estimating the transition parameters with given initial and terminal DNA methylation states, MethylTransition can also calculate the methylation state ratio vector after a cell cycle with given initial DNA methylation states and a set of transition parameters. MethylTransition has been implemented as an R library (Additional file 1: Fig. S1d), and the source code has been released on GitHub (https://github.com/TongjiZhanglab/MethylTransition).

### Performance evaluation of MethylTransition

**Fig. 2:**
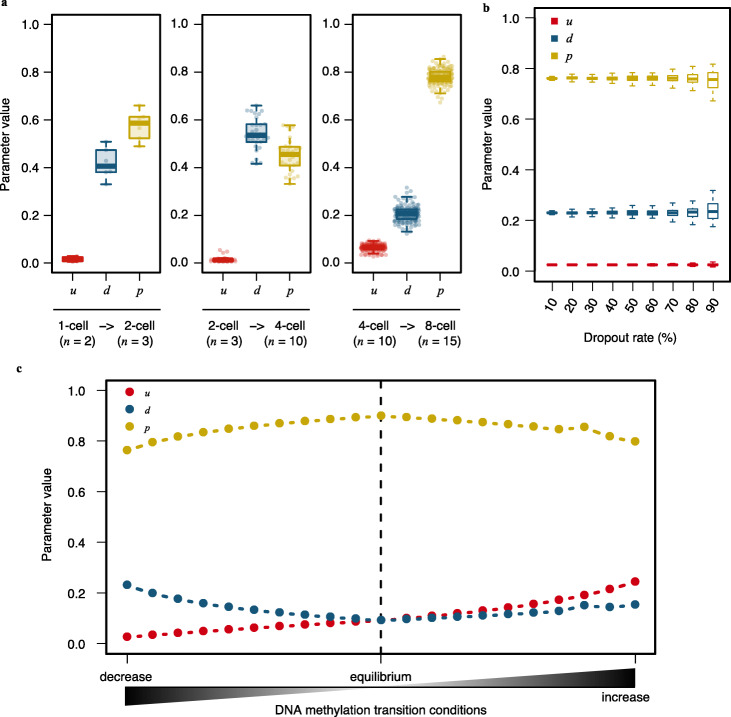
Performance evaluation of MethylTransition. **a** Box plots showing the estimated parameter values from different cell pairs during human first three cell cycles. Each dot represents the parameter value estimated using a pair of scBS-seq data. All available high-quality published scBS-seq data (more than 20% of all CpG sites detected) were used. **b** Box plots showing the robustness of MethylTransition’s parameter estimation for datasets with simulated dropout rates from 10 to 90%. **c** Graph showing the biological meaning of MethylTransition’s parameter estimation for a series of simulated DNA methylation transition conditions (gradually changes from decreasing condition to equilibrium condition and then to increasing condition)

To evaluate the performance of MethylTransition, we used three different strategies. First, we tested the robustness of parameter estimation with respect to cell-to-cell variation. We applied MethylTransition to all available high-quality scBS-seq data at different stages of human pre-implantation embryos [12], and the parameters were estimated for each pair of two cells from the adjacent stages. The variances of parameters estimated by using different cells from the same stage were small (Fig. 2a), confirming the robustness of MethylTransition in parameter estimation with respect to cell-to-cell variation.

Second, we examined the robustness of parameter estimation for different dropout rates of scBS-seq data. We applied MethylTransition to a pair of scBS-seq datasets from two adjacent human pre-implantation stages, the zygote stage and the 2-cell stage, with average promoter DNA methylation levels of 16.8% and 15.0%, respectively. When all 22,056 promoters with sequenced CpG sites in both datasets were used to construct the methylation state ratio vectors, the parameters were estimated as *u* = 0.03, *d* = 0.23, and *p* = 0.76. To evaluate the effect of dropout on parameter estimation, we randomly sampled fractions of the 22,056 promoters to represent dropout rates from 10 to 90% (with 10% as the interval), and the subsets of genes were used to construct the methylation state ratio vectors and then to estimate the parameters (see “Methods” section for details). Across the different dropout rates, the estimated parameters were consistent, with slightly increased standard deviations at high dropout rates (Fig. 2b). Our results demonstrate that the parameter estimation method of MethylTransition is robust even with a high dropout rate of scBS-seq data.

Third, we evaluated whether the estimated parameters could represent the levels of corresponding DNA methylation-modifying activities. The dramatic DNA methylation decrease between the human zygote stage and 2-cell stage was characterized by a higher probability of active demethylation (*d* = 0.23) and a lower probability of methylation maintenance (*p* = 0.76) than that in the equilibrium condition (*d* = 0.09 and *p* = 0.90, respectively), consistent with the findings of previous studies [29]. To confirm the biological meanings of the estimated parameters, we gradually altered the methylation state ratio vector at the 2-cell stage to represent a series of DNA methylation transition conditions (from decrease to equilibrium, then to increase), and the altered vectors were used to estimate the parameters (see the “Methods” section for details). Consistent with our expectations, the probability of active demethylation (*d*) decreased dramatically from DNA methylation decreasing conditions to the equilibrium condition; this decrease in *d* was coupled with rapid increases in the probability of methylation maintenance (*p*) and the probability of de novo methylation (*u*), which clearly increased from the equilibrium condition to increasing conditions (Fig. 2c). To further evaluate the interpretability of parameters, we extended the application of MethylTransition to bulk cell-level BS-seq data with methylation enzymes knockout (see the “Methods” section for details). We collected the BS-seq data in wild-type and two types of enzyme-knockout mouse embryos (*Dnmt1*^−/−^, *Dnmt3a/b*^−/−^) at the E8.5 stage [30]. The *Dnmt1*^−/−^ embryos displayed the smallest *p*, and the *Dnmt3a/b*^−/−^ embryos showed the smallest *u*, which are in agreement with the known function of these DNMTs (Additional file 1: Fig. S2a). In addition, we collected the BS-seq data in wild-type and two types of enzyme-knockout zebrafish embryos (*Tet3*^−/−^, *Tet1/2/3*^−/−^) at 24 h post-fertilization (hpf) [31]. Both *Tet3*^−/−^ embryos and *Tet1/2/3*^−/−^ embryos showed smaller *d* than wild-type embryos, and *Tet1/2/3*^−/−^ embryos showed the smallest *d* (Additional file 1: Fig. S2b). Our results confirmed that the estimated parameters can reflect the levels of DNA methylation-modifying activities.

### Programmed DNA methylation heterogeneity in human pre-implantation embryos

**Fig. 3:**
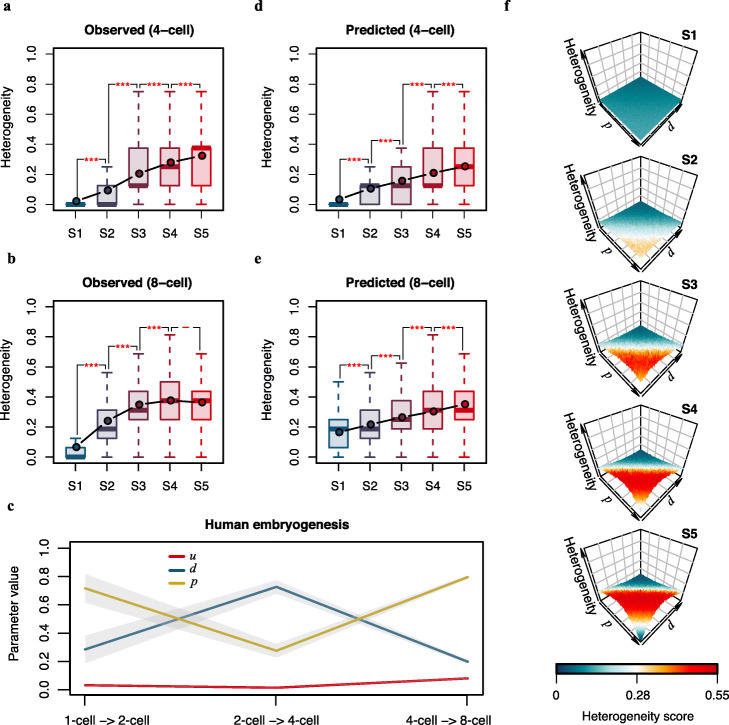
Programmed DNA methylation heterogeneity in human pre-implantation embryos. **a**, **b** Box plots of the observed DNA methylation heterogeneity of promoters in the 4-cell stage (**a**) and 8-cell stage (**b**) during human embryogenesis. Promoters were classified into 5 classes based on their DNA methylation states in the zygote stage. For each box, the point indicates the average DNA methylation heterogeneity of the class. Student’s *t* test was performed for comparisons between adjacent classes (***p value < 0.001; **p value < 0.01; *p value < 0.05; ^-^*p* value > 0.05). The embryo with the largest number of high-quality scBS-seq data (more than 20% of all CpG sites detected) at each stage were used to calculate the DNA methylation heterogeneity. **c** Estimated parameters of the first three cell cycles during human embryogenesis. The lines link the mean values of the parameters in each cell cycle. The gray shaded areas represent the 95% confidence intervals around the mean values. **d**, **e** Box plots of the predicted DNA methylation heterogeneity of promoters in the 4-cell stage (**d**) and 8-cell stage (**e**) during human embryogenesis. Promoters were classified into 5 classes based on their DNA methylation states in the zygote stage. For each box, the point indicates the average DNA methylation heterogeneity of the class. Student’s *t* test was performed for comparisons between adjacent classes (***p value < 0.001). **f** 3D plots of predicted DNA methylation heterogeneity scores at the 8-cell stage for simulated values of *p* and *d* for five classes of promoters. Here, the parameter *u* is a constant (*u* = 0.03). The colors represent the predicted heterogeneity scores

To comprehensively investigate the cause of DNA methylation heterogeneity in early embryogenesis, we reanalyzed scBS-seq data in human pre-implantation embryos. We observed that 31.7~42.6% of the promoters showed DNA methylation state polymorphism among cells within a 4-cell stage embryo, and the percentage increased to 54.4~65.7% for 8-cell stage embryos, confirming that DNA methylation states are quite heterogeneous among cells in pre-implantation embryos [12]. To quantitively measure such heterogeneity, we used public scBS-seq data from a single embryo for each stage and calculated a heterogeneity score for each promoter based on the DNA methylation states from different cells in the same embryo (Additional file 1: Fig. S3; see the “Methods” section for details). When classifying the promoters into five classes based on their DNA methylation states in the zygote stage, we found that the DNA methylation heterogeneity in 4-cell and 8-cell stage embryos was significantly different among classes, while promoters with heavier DNA methylation states in the zygote stage tended to have higher DNA methylation heterogeneity in the 4-cell and 8-cell stages (Fig. 3a, b). Given such an association between the initial methylation states and the DNA methylation heterogeneity at later stages, we hypothesized that the DNA methylation heterogeneity in early embryos might be an intrinsic output of a programmed process following a set of cellular instructions.

To test the above hypothesis, we applied MethylTransition to the first three cell cycles of human embryogenesis using scBS-seq data from a single embryo for each stage. To simplify the calculation, we used all genes’ promoters to estimate the parameters. For each combination of cell pairs between embryos in adjacent stages, the parameters *u*, *d*, and *p* were estimated, with substantial robustness among combinations (Fig. 3c). The highest value of the parameter *d*, i.e., the probability of active demethylation, occurred during the transition between the 2-cell and 4-cell stages, while the highest value of the parameter *p*, i.e., the probability of methylation maintenance, occurred during the transition between the 4-cell and 8-cell stages. The parameter *u*, i.e., the probability of de novo methylation, was consistently low during this process. Given the initial methylation states of the promoters at the zygote stage and the estimated parameters of the first three cell cycles, MethylTransition was applied to predict the DNA methylation heterogeneity for each promoter in the 4-cell and 8-cell stages, respectively (Fig. 3d, e; see the “Methods” section for details). The promoters with heavier DNA methylation states in the zygote stage clearly displayed a higher predicted DNA methylation heterogeneity in the 4-cell and 8-cell stages, consistent with the trends in observed heterogeneity among classes with distinct initial methylation states. Such predictable heterogeneity demonstrates that the DNA methylation heterogeneity in early embryos is largely an intrinsic output of a programmed process with given initial methylation states and a few DNA methylation state transition parameters.

To investigate the impacts of initial methylation states and DNA methylation state transition parameters on this programmed heterogeneity, we calculated the predicted scores of DNA methylation heterogeneity in the 8-cell stage with simulated values of MethylTransition parameters for five classes of promoters with distinct initial methylation states (Fig. 3f). To simplify the simulation, we kept *u* = 0.03, which was supported by the absence of nuclear DNMT3a and DNMT3b during this process [32], and used the same values of *p* or *d* for all three cell cycles. We observed that for any given class, the predicted scores of DNA methylation heterogeneity varied dramatically for different combinations of *p* and *d*, while with a given set of *p* and *d* values, the predicted scores tended to be higher in classes with heavier initial methylation states. In a typical mammalian cell, most promoters are either unmethylated (S1) or fully methylated (S5); in addition, during a typical mammalian cell cycle, the probability of methylation maintenance (*p*) is very high, while the probability of active demethylation (*d*) is quite low [33, 34]. From the simulation, we observed low predicted heterogeneity (score < 0.2) in the S1 and S5 classes for combinations of high *p* (> 0.94) and low *d* (< 0.05) values, indicating that DNA methylation heterogeneity is unlikely to be introduced during the cell cycle in a typical mammalian cell. Unlike typical mammalian cells, the cells in early embryos display clearly higher *d* and lower *p* values, which can intrinsically cause initial methylation state depended on DNA methylation heterogeneity during early embryogenesis. Taken together, our results indicate that the largely programmed DNA methylation heterogeneity in early embryos is a natural result of the unique probabilities of methylation maintenance and active demethylation that occur during this process.

### Association of sequence and epigenetic features with DNA methylation heterogeneity

**Fig. 4:**
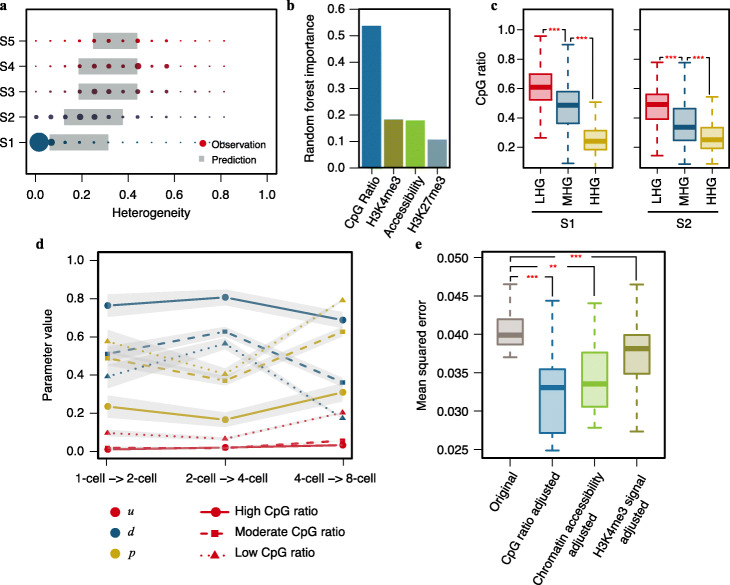
Impacts of sequence and epigenetic features on DNA methylation heterogeneity. **a** Differences between the observed and predicted DNA methylation heterogeneity scores in human 8-cell stage embryos. Promoters were classified into 5 classes based on their DNA methylation states in the zygote stage. The size of each solid circle represents the number of promoters. Each gray box shows the 1st and 3rd quartiles of the predicted heterogeneity scores in each class. **b** Bar plot of the importance of sequence and epigenetic features for DNA methylation heterogeneity at the 8-cell stage, as determined by random forest analysis. The features include CpG ratio, chromatin accessibility, and H3K4me3 and H3K27me3 levels at the 8-cell stage. **c** Box plots of the differences in CpG ratio between different categories of promoters in the S1 and S2 classes. The promoters in the S1 and S2 classes were divided into three categories: higher-heterogeneity gene promoters (HHGs; with observed heterogeneity higher than the 3rd quantile of the predicted heterogeneity score of the class), model-predictable heterogeneity gene promoters (MHGs), and lower-heterogeneity gene promoters (LHGs; with observed heterogeneity lower than the 1st quantile of the predicted heterogeneity score of the class). Student’s *t* test was performed for comparisons between adjacent categories (***p value < 0.001). **d** Estimated parameters for different groups of promoters with distinct CpG ratios during the first three cell cycles of human embryogenesis. The promoters were grouped into three groups with high (≥ 0.6), moderate (between 0.4 and 0.6), or low (< 0.4) CpG ratios. The lines link the mean values of the parameters for each cell cycle. The gray shaded areas represent the 95% confidence intervals around the mean values. Red indicates the parameter *u*, blue indicates the parameter *d*, and yellow indicates the parameter *p*. **e** Box plots of the differences in the mean squared error between the original prediction obtained using uniform sets of *u*, *d*, and *p* for all promoters and three conditional predictions based separately on CpG ratio grouping, chromatin accessibility grouping, and H3K4me3 signal grouping. The mean squared error was calculated as the average squared difference between the predicted methylation heterogeneity score and the observed score. Student’s *t* test was performed for comparisons between the original prediction and each of the conditional predictions (***p value < 0.001; **p value < 0.01)

Although the DNA methylation heterogeneity in early embryos was largely predictable by MethylTransition with a few parameters, some promoters showed substantially higher or lower scores of observed methylation heterogeneity than predicted, especially promoters in the S1 and S2 classes (Fig. 4a, Additional file 1: Fig. S4a), indicating that additional features may have significant impacts on the DNA methylation heterogeneity of promoters in these classes. In order to investigate which sequence or epigenetic features may strongly associate with DNA methylation heterogeneity, we performed random forest analysis to identify relevant features from sequence patterns (CpG ratio), chromatin accessibility, and histone modification patterns (H3K4me3, H3K27me3) in human pre-implantation embryos (see the “Methods” section for details). Among these features, the CpG ratio showed much greater relevance to promoter DNA methylation heterogeneity than other features. Both chromatin accessibility and H3K4me3 signals showed moderate relevance (Fig. 4b). For the S1 and S2 classes, the promoters with lower heterogeneity scores than predicted showed significantly higher CpG ratio, chromatin accessibility, and H3K4me3 signals than those with heterogeneity scores similar to the predicted scores (Fig. 4c, Additional file 1: Fig. S4b, c), suggesting that the limited performance of MethylTransition for the S1 and S2 classes is likely due to these sequence and epigenetic features. To examine whether DNA methylation state transition parameters are variable among promoters with distinct CpG ratios, we divided promoters into three groups with high (≥ 0.6), moderate (between 0.4 and 0.6), or low (< 0.4) CpG ratio and applied MethylTransition to the first three cell cycles of human embryogenesis based on the three groups of promoters separately. The sets of the estimated parameters *u*, *d*, and *p* were different among groups of promoters with distinct CpG ratios (Fig. 4d). The group of promoters with a high CpG ratio showed lower values of *u* and *p*, together with higher values of *d*, than the other two groups, consistent with the findings of previous studies that CpG islands tend to be protected from DNA methylation [35]. We further predicted the DNA methylation heterogeneity for each promoter in the 8-cell stage using parameter values estimated based on CpG ratio grouping, and the predicted heterogeneity showed a significantly smaller mean squared error to the observed than the original prediction obtained using uniform *u*, *d*, and *p* values for all promoters (Fig. 4e). In addition to assessing DNA methylation state transition parameters among promoters with distinct CpG ratio, we also calculated the parameters among promoters with distinct chromatin accessibility or distinct H3K4me3 signals (Additional file 1: Fig. S4d, e). The heterogeneity predicted using parameters estimated based on chromatin accessibility or H3K4me3 signal grouping also showed a significantly smaller mean squared error to the observed than the original prediction (Fig. 4e). Taken together, our results demonstrate that the features of CpG ratio, chromatin accessibility, and distinct H3K4me3 signals are strongly associated with DNA methylation heterogeneity by diverse DNA methylation state transition parameters.

### Potential regulatory effects of DNA methylation heterogeneity

**Fig. 5:**
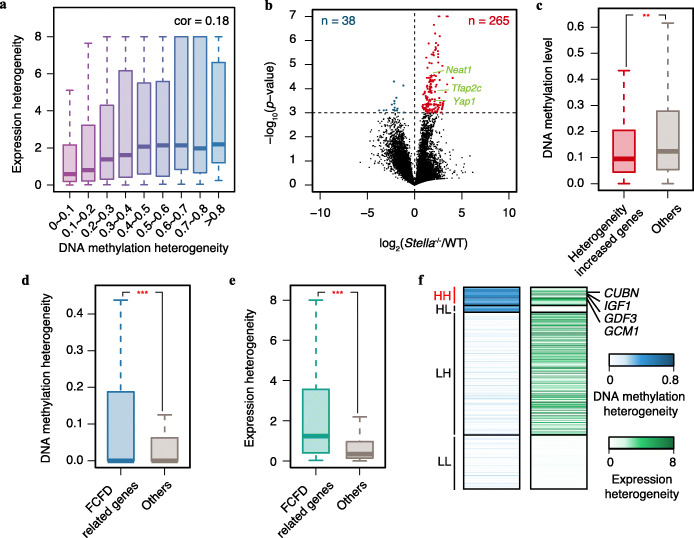
Potential regulatory effects of DNA methylation heterogeneity. **a** Box plots of the relationship between DNA methylation heterogeneity and expression heterogeneity in human 8-cell stage embryos. The Pearson correlation coefficient of the two heterogeneity scores was 0.18. **b** Volcano plot comparing the gene expression heterogeneity between mouse *Stella*^−/−^ embryos and mouse wild-type (WT) embryos at the 8-cell stage. The *x*-axis represents the log_2_-transformed fold change, and the *y*-axis represents the log_10_-transformed *p* value. A total of 265 genes had significantly higher expression heterogeneity scores in *Stella*^−/−^ embryos than in WT embryos, while 38 genes had significantly lower expression heterogeneity scores in *Stella*^−/−^ embryos than in WT embryos. Three genes reported to be related to cell fate determination during mouse embryogenesis are indicated in green. **c** Box plots comparing the DNA methylation levels in mouse zygotes between genes with increased expression heterogeneity and other genes. Student’s *t* test was performed (**p value < 0.01). **d**, **e** Box plots of the DNA methylation heterogeneity (**d**) and expression heterogeneity (**e**) in human 8-cell stage embryos between first cell fate determination (FCFD)-related genes and other genes. Student’s *t* test was performed for comparisons (***p value < 0.001). **f** Heatmap of the DNA methylation heterogeneity scores (blue) and expression heterogeneity scores (green) for FCFD-related genes. The FCFD-related genes were classified into four groups based on their DNA methylation and expression heterogeneity scores at the 8-cell stage (HH: DNA methylation heterogeneity > 0.3 and expression heterogeneity > 0.5; HL: DNA methylation heterogeneity > 0.3 and expression heterogeneity ≤ 0.5; LH: DNA methylation heterogeneity ≤ 0.3 and expression heterogeneity > 0.5; and LL: DNA methylation heterogeneity ≤ 0.3 and expression heterogeneity ≤ 0.5). Within the HH group, three reported ICM markers (*GDF3*, *CUBN*, and *IGF1*) and a TE marker (*GCM1*) are indicated

As DNA methylation at promoters plays important roles in gene transcriptional regulation, we hypothesized that the largely programmed DNA methylation heterogeneity at promoters can affect the heterogeneity of gene expression. To test this hypothesis, we first calculated the correlation between promoter DNA methylation heterogeneity scores and expression heterogeneity scores in human 8-cell stage embryos (see the “Methods” section for details), and the two types of heterogeneity scores indeed showed a positive correlation (cor = 0.18; Fig. 5a). Next, we investigated the temporal association between promoter DNA methylation heterogeneity and expression heterogeneity. The genes with high DNA methylation heterogeneity promoters at the 4-cell stage showed significantly larger expression heterogeneity increasement from that stage to the 8-cell stage than other genes (Additional file 1: Fig. S5a). Differently, the genes with high expression heterogeneity at the 4-cell stage showed similar promoter DNA methylation heterogeneity increasement from that stage to the 8-cell stage with other genes (Additional file 1: Fig. S5b), which implied that DNA methylation heterogeneity at promoters might affect the heterogeneity of the gene expression. Due to restrictions preventing the editing of human embryos, we then used mouse embryos to further clarify the effects of DNA methylation heterogeneity on expression heterogeneity. Similar to the case in human embryos, the DNA methylation heterogeneity that emerged during mouse early embryogenesis was also largely determined by the initial DNA methylation state at the zygote stage (Additional file 1: Fig. S6). In this study, we generated single-cell RNA-seq data for cells in the 8-cell stage from *Stella*^−/−^ mouse embryos, which have been reported to have much higher DNA methylation levels than normal embryos [36]. Upon comparing the expression heterogeneity scores of *Stella*^−/−^ mouse embryos to those of normal embryos, we identified 6.97-fold more genes with significantly elevated heterogeneity scores in *Stella*^−/−^ embryos than genes with reduced scores including genes encoding reported lineage-determining factors of ICM and TE (*Neat1* [9], *Tfap2c* [37], *Yap1* [38, 39]) (Fig. 5b). The genes with elevated expression heterogeneity scores in *Stella*^−/−^ embryos showed significantly lower initial DNA methylation levels at promoters in the zygote stage in normal mouse embryos than other genes (Fig. 5c), indicating that increases in initial DNA methylation levels can increase the expression heterogeneity in later stages, especially for genes with initially minimally methylated promoters. Considering that promoters with heavier DNA methylation in the zygote stage tended to have higher DNA methylation heterogeneity in later stages, the increased expression heterogeneity in *Stella*^−/−^ embryos might be mediated by the largely programmed DNA methylation heterogeneity at promoters.

We further hypothesized that the largely programmed DNA methylation heterogeneity at promoters might contribute to the first cell fate determination. To test this hypothesis, among 7471 expressed genes in ICM or TE, we defined 899 ICM- or TE-specific genes as the list of genes related to the first cell fate determination in humans (see the “Methods” section for details). At the 8-cell stage, that list of genes showed significantly higher DNA methylation heterogeneity at promoters, together with higher expression heterogeneity, than other expressed genes (Fig. 5d, e), indicating that the separation between the ICM and TE could at least partially be explained by the emerged expression heterogeneity and by largely programmed DNA methylation heterogeneity at promoters. Among the list of genes related to the first cell fate determination, 64 genes showed both high DNA methylation heterogeneity at promoters and expression heterogeneity at the 8-cell stage (Fig. 5f and Additional file 2: Table S1), including the known ICM marker genes *GDF3* [40], *CUBN* [41], and *IGF1* [42] and the known TE marker gene *GCM1* [43]. The clear association between DNA methylation heterogeneity and the first cell fate determination suggests that consideration of DNA methylation heterogeneity may provide clues for identifying driving factors of driving cell fate determination during human pre-implantation embryogenesis.

## Discussion

Although expression and epigenetic heterogeneity occurring before the first cell fate determination in mammalian early embryogenesis has been suggested to be a determinate result of a program [10–12] (i.e., a process with initial states and a set of cellular instructions), to the best of our knowledge, no studies have been designed to illustrate such a program. In this study, we took advantage of the quantitative features of scBS-seq data to elucidate the set of instructions causing DNA methylation heterogeneity during early embryogenesis by building MethylTransition, a mathematical model that enables characterization of the DNA methylation state transition during single or a few cell cycles. The results of this study clearly demonstrate that DNA methylation heterogeneity at promoters in early mammalian embryos is largely an intrinsic output of a programmed process with given initial methylation states and a few DNA methylation state transition parameters. It should be clarified that the cell cycle itself does not naturally result in DNA methylation heterogeneity. Calculations based on MethylTransition showed that DNA methylation heterogeneity is unlikely to be introduced during the cell cycle in typical adult mammalian cells, which have different initial methylation state distributions and DNA methylation state transition parameters than cells in early mammalian embryos. This study illustrates that the largely programmed DNA methylation heterogeneity in early mammalian embryos is a determinate result of the unique probabilities of methylation maintenance and active demethylation that occur during early embryogenesis.

The process of the first cell fate determination in early mammalian embryos is associated with distinct expression and epigenetic patterns between ICM and TE cells [6, 7]. This study showed a clear association between DNA methylation heterogeneity and the first cell fate determination, and the results suggest that the expression heterogeneity that emerges before the first cell fate determination could be at least partially caused by largely programmed DNA methylation heterogeneity. In addition to DNA methylation, multiple layers of factors participate in transcriptional regulation. A previous model study based on mouse embryogenesis data proposed that the first cell fate determination-related expression heterogeneity is initiated by random segregation at each cleavage division and strengthened by competing transcriptional circuits [5]. In human embryos, the major phase of zygotic genome activation begins at the 8-cell stage, much later than in mouse embryos (in which it occurs at the 2-cell stage) [44], indicating that humans have a larger time window for the accumulation of DNA methylation heterogeneity before the large-scale transcription begins. As a supplement to that model study, this study suggests that the initial source of expression heterogeneity in human embryos could be at least partially mediated by the largely programmed DNA methylation heterogeneity at promoters. We suspect that consideration of DNA methylation heterogeneity could provide valuable clues for identifying driving factors of the first cell fate determination during human pre-implantation embryogenesis.

Unlike other mathematical models, MethylTransition was designed to characterize the methylation state changes that occur during one or a few cell cycles at single-cell resolution, and its application could be extended to other biological processes with dramatic DNA methylation changes, such as oogenesis [45]. Despite its unique features, MethylTransition has two technical limitations. First, due to the high dropout rates of public scBS-seq data, MethylTransition relies on the assumption that the activity levels of DNA methylation-modifying enzymes in a cell are constant across the genome, which is inconsistent with numerous reports that both sequence and epigenetic features affect the activity of these enzymes [25, 46–49]. This limitation could be partially resolved by applying this framework to a subset of promoters with similar sequence or epigenetic features. Second, MethylTransition assumes that de novo methylation, methylation maintenance, and active demethylation are three independent events. However, this may not always be true, as crosstalk between de novo methylation and methylation maintenance has been reported [50–52]. Those studies indicated that the functions of the DNA methylation-modifying enzymes are complex and not independent. Furthermore, some factors, for example, Stella, have distinct roles in the modulation of DNA methylation at different mouse development stages [36, 53]. Although the parameters of MethylTransition integrated the function of various enzymes to represent the overall levels of DNA methylation-modifying activities, incorporating the crosstalk of those activities may further improve the interpretability of this model. Future versions of MethylTransition could be extended to consider the conditional probabilities among the three DNA methylation-modifying activities.

## Conclusions

To quantitatively characterize the DNA methylation state transition during mammalian early embryogenesis, we presented MethylTransition, a novel DNA probabilistic model for characterizing methylation changes during one or a few cell cycles at single-cell resolution. By applying MethylTransition to scBS-seq data during human pre-implantation embryogenesis, we elucidated that the DNA methylation heterogeneity that emerges at promoters is a determinate result of the unique probabilities of methylation maintenance and active demethylation that occur during early embryogenesis. This study further suggested that consideration of programmed DNA methylation heterogeneity provides valuable clues for identifying driving factors of the first cell fate determination.

## Methods

### Computational framework of MethylTransition

We used *1* as a symbol to represent methylated CpG dinucleotides and *0* to represent unmethylated CpG dinucleotides. Thus, the DNA methylation state for a CpG dyad in one chromosome could be one of the following types: unmethylated (*0-0*), hemi-methylated (*0-1* or *1-0*), or fully methylated (*1-1*). The calculated ratios of CpG dyads in these four methylation states are *x*_1_, *x*_2_, *x*_3_, and *x*_4_, and the ratio vector *M*_*s*_ is:

1 $$ {M}_s=\left(\begin{array}{c}{x}_1\\ {}{x}_2\\ {}{x}_3\\ {}{x}_4\end{array}\right) $$

MethylTransition relies on the assumption that changes in the DNA methylation state at a CpG site across a single cell cycle occur in three steps. In the first step, subsequent to the passive demethylation of DNA with DNA replication in the cell, the ratio vector *M*_*s*_ changes to *M*_*r*_, which can be represented as:

2 $$ {T}_{sr}=\left(\begin{array}{cccc}1& a& \left(1-a\right)& 0\\ {}0& \left(1-a\right)& 0& \left(1-a\right)\\ {}0& 0& a& a\\ {}0& 0& 0& 0\end{array}\right) $$

3 $$ {M}_r={T}_{sr}\bullet {M}_s $$

where *a* represents the probability of parental DNA in one of two daughter strands and is 0.5 for the symmetric division. *M*_*r*_ is the ratio vector after DNA replication.

In the second step, the DNA methylation state in a CpG dyad can be altered by DNMTs or by DNA demethylases. We denoted the probability of maintaining methylation at a hemi-methylated CpG site as *p*, the probability of de novo methylation at a CpG site as *u*, and the probability of active demethylation at a methylated CpG site as *d*. The change in DNA methylation under the actions of various enzymes can be expressed as:

4 $$ {T}_{re}=\left(\begin{array}{cccc}\left(1-u\right)\times \left(1-u\right)& \left(1-u\right)\times \left(1-p\right)\times d& d\times \left(1-u\right)\times \left(1-p\right)& d\times d\\ {}u\times \left(1-u\right)& \left(1-p\right)\times \left(1-u\right)\times \left(1-d\right)& d\times \left(u+p-u\times p\right)& \left(1-d\right)\times d\\ {}\left(1-u\right)\times u& \left(u+p-u\times p\right)\times d& \left(1-d\right)\times \left(1-u\right)\times \left(1-p\right)& d\times \left(1-d\right)\\ {}u\times u& \left(u+p-u\times p\right)\times \left(1-d\right)& \left(1-d\right)\times \left(u+p-u\times p\right)& \left(1-d\right)\times \left(1-d\right)\end{array}\right) $$

5 $$ {M}_e={T}_{re}\bullet {M}_r $$

where *M*_*e*_ represents the ratio vector after actions have been exerted by various enzymes.

In the third step, for diploid organisms, the observed DNA methylation state of a CpG site in a single cell is the combination of the DNA methylation states from both homologous chromosomes. Such combination results in five possible DNA methylation states at each CpG site: unmethylated (labeled S1), quarter-methylated (S2), half-methylated (S3), three-quarter-methylated (S4), and fully methylated (S5). These states can be experimentally measured, and the ratios of CpG sites in these five methylation states (*S*_1_, *S*_2_, *S*_3_, *S*_4_, and *S*_5_) can be calculated. Thus, the DNA methylation state changes from mother cell to daughter cell are represented by:

6 $$ {T}_A=\left(\begin{array}{ccccc}{x}_{11}& {x}_{12}& {x}_{13}& {x}_{14}& {x}_{15}\\ {}{x}_{21}& {x}_{22}& {x}_{23}& {x}_{24}& {x}_{25}\\ {}{x}_{31}& {x}_{32}& {x}_{33}& {x}_{34}& {x}_{35}\\ {}{x}_{41}& {x}_{42}& {x}_{43}& {x}_{44}& {x}_{45}\\ {}{x}_{51}& {x}_{52}& {x}_{53}& {x}_{54}& {x}_{55}\end{array}\right) $$

7 $$ \left(\begin{array}{c}{S_1}^{\prime}\\ {}{S_2}^{\prime}\\ {}{S_3}^{\prime}\\ {}{S_4}^{\prime}\\ {}{S}_5\end{array}\right)={T}_A\bullet \left(\begin{array}{c}{S}_1\\ {}{S}_2\\ {}{S}_3\\ {}{S}_4\\ {}{S}_5\end{array}\right) $$

where *S*_1_^′^, *S*_2_^′^, *S*_3_^′^, *S*_4_^′^, and *S*_5_^′^ represent the ratios of CpG sites in the five DNA methylation states in a daughter cell, and *x*_*ij*_ indicates the probability that the DNA methylation states are changed from state *j* in the mother cell to state *i* in the daughter cell. The element in the *i*th row and the *j*th column of the probability matrix *T*_*re*_ ∙ *T*_*sr*_ described above is denoted as *t*_*ij*_. The relationships between *x*_*ij*_ and *t*_*ij*_ are calculated as follows:

8 $$ {\displaystyle \begin{array}{c}{x}_{11}={t}_{11}\times {t}_{11}\\ {}{x}_{12}=\frac{1}{4}\times \left({t}_{11}\times {t}_{12}+{t}_{11}\times {t}_{13}+{t}_{12}\times {t}_{11}+{t}_{13}\times {t}_{11}\right)\\ {}{x}_{13}=\frac{1}{6}\times \left({t}_{11}\times {t}_{14}+{t}_{12}\times {t}_{12}+{t}_{12}\times {t}_{13}+{t}_{13}\times {t}_{12}+{t}_{13}\times {t}_{13}+{t}_{14}\times {t}_{11}\right)\\ {}{x}_{14}=\frac{1}{4}\times \left({t}_{12}\times {t}_{14}+{t}_{13}\times {t}_{14}+{t}_{14}\times {t}_{12}+{t}_{14}\times {t}_{13}\right)\\ {}{x}_{15}={t}_{14}\times {t}_{14}\\ {}\dots \dots \\ {}{x}_{51}={t}_{41}\times {t}_{41}\\ {}{x}_{52}=\frac{1}{4}\times \left({t}_{41}\times {t}_{42}+{t}_{41}\times {t}_{43}+{t}_{42}\times {t}_{41}+{t}_{43}\times {t}_{41}\right)\\ {}{x}_{53}=\frac{1}{6}\times \left({t}_{41}\times {t}_{44}+{t}_{42}\times {t}_{42}+{t}_{42}\times {t}_{43}+{t}_{43}\times {t}_{42}+{t}_{43}\times {t}_{43}+{t}_{44}\times {t}_{41}\right)\\ {}{x}_{54}=\frac{1}{4}\times \left({t}_{42}\times {t}_{44}+{t}_{43}\times {t}_{44}+{t}_{44}\times {t}_{42}+{t}_{44}\times {t}_{43}\right)\\ {}{x}_{55}={t}_{44}\times {t}_{44}\end{array}} $$

Based on the above three steps, the changes in DNA methylation states during one cell cycle can be represented by the above probability matrix *T*_*A*_ with a few parameters. Then scBS-seq data are used to infer the ratio of each DNA methylation state within a single cell. To imitate the DNA methylation state transition before and after the cell cycle, the state transition frequency matrix (*T*_*B*_) is calculated using scBS-seq data of a cell from the early stage (mother cell) and a cell from the adjacent late stage (daughter cell) as follows:

9 $$ {T}_B=\left(\begin{array}{ccccc}{o}_{11}& {o}_{12}& {o}_{13}& {o}_{14}& {o}_{15}\\ {}{o}_{21}& {o}_{22}& {o}_{23}& {o}_{24}& {o}_{25}\\ {}{o}_{31}& {o}_{32}& {o}_{33}& {o}_{34}& {o}_{35}\\ {}{o}_{41}& {o}_{42}& {o}_{43}& {o}_{44}& {o}_{45}\\ {}{o}_{51}& {o}_{52}& {o}_{53}& {o}_{54}& {o}_{55}\end{array}\right) $$

where *o*_*ij*_ is the observed frequency at which the DNA methylation state is changed from state *j* in the mother cell to state *i* in the daughter cell.

MethylTransition estimates the parameters *u*, *d*, and *p* by minimizing the difference between the transition probability matrix *T*_*A*_ and the calculated transition frequency matrix *T*_*B*_. We defined a cost function as the square of the Euclidean distance between *T*_*A*_ and *T*_*B*_ [28]:

10 $$ J\left(u,d,p\right)=\sum \limits_{i=1}^5\sum \limits_{j=1}^5{\left({o}_{i,j}-{x}_{i,j}\right)}^2 $$

The best parameter was obtained by minimizing *J*(*u*, *d*, *p*). Although it is unrealistic to expect an algorithm to find global minima, many numerical optimization techniques can be applied to find local minima. According to the definition of probability, the parameters *u*, *d*, and *p* range from 0 to 1. Considering the reliability and efficiency of the algorithm, we used the Newton-Raphson method as the optimization algorithm.

11 $$ \left(\hat{u},\hat{d},\hat{p}\right)={\mathrm{argmin}}_{u,d,p}J\left(u,d,p\right) $$

As the estimates of parameters with different initial values in the Newton-Raphson method are distributed around the true value, MethylTransition randomly selects three numbers in the range of 0 to 1 as the initial values of *u*, *d*, and *p* to estimate the parameters and repeats the analysis 100 times. The parameters that minimize the cost function value are prioritized.

### Calculation of the DNA methylation state ratio vector

We assigned a DNA methylation state *S* to each gene promoter region (± 2 kb around the transcription start site (TSS)) for a given cell, as follows:

12 $$ S=\left\{\begin{array}{c}\mathrm{S}1,\mathrm{if}\ m\le 0.125\\ {}\mathrm{S}2,\mathrm{if}\ 0.125\le m<0.375\\ {}\mathrm{S}3,\mathrm{if}\ 0.375\le m<0.625\\ {}\mathrm{S}4,\mathrm{if}\ 0.625\le m<0.875\\ {}\mathrm{S}5,\mathrm{if}\ m\ge 0.875\\ {}\mathrm{NA},\mathrm{if}\ m\ \mathrm{is}\ \mathrm{not}\ \mathrm{available}\end{array}\right. $$

where *m* is the average methylation level of the promoter. To avoid the missing value problem of scBS-seq data, the promoters with state as NA are removed from the parameter estimation. The DNA methylation state ratio vector contains 5 items representing the fractions of the promoters assigned as S1, S2, S3, S4, and S5, and the vector is used to present the overall pattern of methylation states for a given cell.

### Performance evaluation for the robustness of subsampling

We calculated the DNA methylation state vectors of two adjacent human pre-implantation stages based on two scBS-seq datasets (GSM2481558 and GSM2481533). To evaluate the effects of dropout on parameter estimation, we used a bootstrapping approach. Briefly, we randomly sampled fractions of 22,056 promoters to represent the dropout rates from 10 to 90% (with 10% as the interval) with 100 rounds of replacements for each dropout rate. In each sampling, the selected promoters were used to construct a methylation state ratio vector and a transition frequency matrix, which were used to estimate a set of parameters.

### Performance evaluation for biological meanings

We used the original transition frequency matrix calculated based on the two scBS-seq datasets (GSM2481558 and GSM2481533) as the decreased methylation transition condition, and we used an identity matrix to represent the equilibrium condition. To simulate a gradual change in transition conditions from decreasing to equilibrium, we generated 9 transition frequency matrixes to represent the linear switch from the original transition frequency matrix to an identical matrix with equal intervals. We further transposed and scaled the original transition frequency matrix as the increased transition condition. To simulate a gradual change in transition conditions from equilibrium to increasing, we generated 9 transition frequency matrixes to represent the linear switch from the identical matrix to the transposed and scaled matrix with equal intervals. We estimated the sets of parameters based on the original transition frequency matrix, the identical matrix, the transposed and scaled matrix, and 18 generated transition frequency matrixes.

### Extension of MethylTransition on bulk-cell BS-seq data

Each high coverage CpG site (coverage > 4) in a given bulk cell BS-seq data was assigned a DNA methylation state *S*, as follows:

13 $$ S=\left\{\begin{array}{c}\mathrm{S}1,\mathrm{if}\ {m}_b\le 0.125\\ {}\mathrm{S}2,\mathrm{if}\ 0.125\le {m}_b<0.375\\ {}\mathrm{S}3,\mathrm{if}\ 0.375\le {m}_b<0.625\\ {}\mathrm{S}4,\mathrm{if}\ 0.625\le {m}_b<0.875\\ {}\mathrm{S}5,\mathrm{if}\ {m}_b\ge 0.875\\ {}\mathrm{NA},\mathrm{if}\ {m}_b\ \mathrm{is}\ \mathrm{not}\ \mathrm{available}\end{array}\right. $$

where *m*_*b*_ is the methylation level of the CpG site. The sites with state as NA are removed from the parameter estimation. The transition frequency matrix was calculated using two paired BS-seq data. We assigned an approximate number of cell cycles based on the knowledge of development timing (*n* = 5 for 8-cell to E8.5 mouse embryo and *n* = 10 for epiboly to 24 hpf zebrafish embryo). The logarithm transformation of the transition frequency matrix was used to estimate the parameters during multiple cell cycles, with the assumption that the parameters of each cell cycle are consistent.

### Calculation of DNA methylation heterogeneity and expression heterogeneity

The DNA methylation heterogeneity of a given promoter in a pre-implantation embryo represents the promoter’s polymorphism of methylation states across cells in the same embryo. For each promoter, we calculated the proportions of cells belonging to each of the five methylation states. Then, we calculated the inequality of the methylation state distribution combined with the Gini index to quantitively measure the heterogeneity (Additional file 1: Fig. S3). If *x*_*i*_ is the proportion of the methylation state *i*, and there are *n* states (*n* = 5), then the DNA methylation heterogeneity *H*_methyl_ is given by:

14 $$ {H}_{\mathrm{methyl}}=1-\frac{\sum_{i=1}^n{\sum}_{j=1}^n\left|{x}_i-{x}_j\right|}{2n{\sum}_{i=1}^n{x}_i} $$

The expression heterogeneity of a given gene in a pre-implantation embryo represents the polymorphism of gene expression across cells. For each gene, we calculated the expression heterogeneity *H*_expr_ as the squared coefficient of variation (CV^2^) of the expression level across the cells in the same embryo.

### Prediction of DNA methylation heterogeneity

With given parameters (*u*, *d*, *p*) and an initial DNA methylation state ratio vector (*M*_*i*_), the DNA methylation transition matrix (*T*_(*u*, *d*, *p*)_) and the terminal DNA methylation state ratio vector (*M*_*t*_) can be calculated by the equations mentioned in the “Computational framework of MethylTransition” section. For each promoter, the probability that its DNA methylation state at the zygote stage will change to any of the five states in the 2-cell stage can be calculated. Then, a random sampling method (based on the R function *sample()*) was used to assign each promoter a state in each of the 2-cell stage cells based on the calculated probability and the initial DNA methylation state in the zygote stage cell. For different cells in the same stage, different random seeds were used. The procedures for the prediction of the state transition from the 2-cell stage to the 4-cell stage and those for prediction of the state transition from the 4-cell stage to the 8-cell stage were the same. For each promoter, the DNA methylation heterogeneity *H*_methyl_ was calculated as mentioned above with the predicted DNA methylation states for each cell in 4-cell or 8-cell stage embryos.

### Identification of relevant features of DNA methylation heterogeneity

The CpG ratio was calculated for each promoter, as previously described [54]. The chromatin accessibility on each promoter was also calculated using a published assay for transposase-accessible chromatin using sequencing (ATAC-seq) data for human 8-cell stage embryos [55]. The H3K4me3 and H3K27me3 signals on each promoter were derived from published ChIP-seq data from human 8-cell stage embryos [56]. Based on the differences between the observed and predicted DNA methylation heterogeneity scores for the gene promoters, the promoters in classes S1 and S2 were divided into three categories: higher-heterogeneity gene promoters (HHGs; with observed heterogeneity higher than the 3rd quantile of the predicted heterogeneity score of the class), model-predictable heterogeneity gene promoters (MHGs), and lower-heterogeneity gene promoters (LHGs; with observed heterogeneity lower than the 1st quantile of the predicted heterogeneity score of the class). With the values for the above sequence and epigenetic features and the category labels, we performed random forest analysis using the *RandomForestRegressor* function in the Python package *sklearn* [57] to evaluate the importance of each feature to the DNA methylation heterogeneity.

### Identification of human first cell fate determination-related genes

We first excluded genes that were not expressed in blastocysts (genes with FPKM values less than 1 in ICM and TE cells). We then identified differentially expressed genes between ICM and TE cells using DESeq2 with a threshold of *p* value < 0.05 and fold change > 4. These differentially expressed genes were considered as the genes related to human first cell fate determination.

### Single-cell RNA-seq library generation and sequencing of *Stella*^*−/−*^ mouse embryos

*Stella*^*−/−*^ mice have been described previously [36]. RNA was isolated from each single cell of two 8-cell stage *Stella*^*−/−*^ embryos with the Smart-seq2 protocol. To generate RNA sequencing libraries, a KAPA Hyper Prep Kit was used following the manufacturer’s instructions. The adapters used were from a KAPA Single-Indexed Adapter Kit. Paired-end 150-bp sequencing was further performed on a HiSeq X Ten platform (Illumina) at Novogene. The animal experimental procedures were performed according to the Tongji University Guide for the use of laboratory animals.

## Supplementary Information

**Additional file 1:** **Fig. S1.** Computational framework of MethylTransition. **Fig. S2.** Estimated parameters in DNA methylation enzyme knockout samples. **Fig. S3.** Schematic workflow of the calculation of DNA methylation heterogeneity. **Fig. S4.** Impacts of epigenetic features on DNA methylation heterogeneity. **Fig. S5.** The temporal association between promoter DNA methylation heterogeneity and expression heterogeneity. **Fig. S6.** Programmed DNA methylation heterogeneity in mouse pre-implantation embryos.

**Additional file 2:** **Table S1.** A total of 64 genes showed both high DNA methylation heterogeneity at promoters and expression heterogeneity in human 8-cell stage embryos.

**Additional file 3.** Review history.

## Data Availability

The raw sequence data generated in this manuscript have been deposited in the ArrayExpress database at EMBL-EBI (https://www.ebi.ac.uk/arrayexpress) under accession number E-MTAB-9360 [58, 59] and the Genome Sequence Archive (GSA) in BIG Data Center (https://bigd.big.ac.cn/gsa) with accession number CRA002264 [60]. The public scBS-seq data of human early embryos used in this paper are available at Gene Expression Omnibus (GEO) under accession GSE81233 [12]. The public COOL-seq data of human early embryos used in this paper are available at GEO under accession GSE100272 [16]. The public scRNA-seq data of human early embryos used in this paper are available at GEO under accession GSE36552 [61] and GSE71318 [62]. The public ChIP-seq data of human early embryos used in this paper are available at GEO under accession GSE124718 [56]. The public ATAC-seq data of human early embryos used in this paper are available at GEO under accession GSE101571 [55]. The public COOL-seq data of mouse early embryos used in this paper are available at GEO under accession GSE78140 [15]. The public scRNA-seq data of mouse early embryos used in this paper are available at GEO under accession GSE45719 [63], GSE65160 [64], and GSE98150 [65]. The public BS-seq data of mouse embryos with methylation enzyme knockout used in this paper are available at GEO under accession GSE130735 [30]. The public BS-seq data of zebrafish embryos with methylation enzyme knockout used in this paper are available at GEO under accession GSE68087 [66]. MethylTransition is an open-source R package under GNU General Public License v3.0, and it is available at https://github.com/TongjiZhanglab/MethylTransition [26] and 10.5281/zenodo.4075746 [67]. The data analysis in this manuscript was conducted using several custom scripts, all available at https://github.com/TongjiZhanglab/MethylTransition_DataAnalysis.
