# Supplementary material for: A DNA methylation state transition model reveals the programmed epigenetic heterogeneity in human pre-implantation embryos
Source: Genome Biol. 2020 Nov 16;21:277. doi: 10.1186/s13059-020-02189-8 (PMC7667739; doi:10.1186/s13059-020-02189-8)
Supplement: Supplementary file 1 — Additional file 1: Fig. S1. Computational framework of MethylTransition. Fig. S2. Estimated parameters in DNA methylation enzyme knockout samples. Fig. S3. Schematic workflow of the calculation of DNA methylation heterogeneity. Fig. S4. Impacts of epigenetic features on DNA methylation heterogeneity. Fig. S5. The temporal association between promoter DNA methylation heterogeneity and expression heterogeneity. Fig. S6. Programmed DNA methylation heterogeneity in mouse pre-implantation embryos. [file 13059_2020_2189_MOESM1_ESM.pdf]

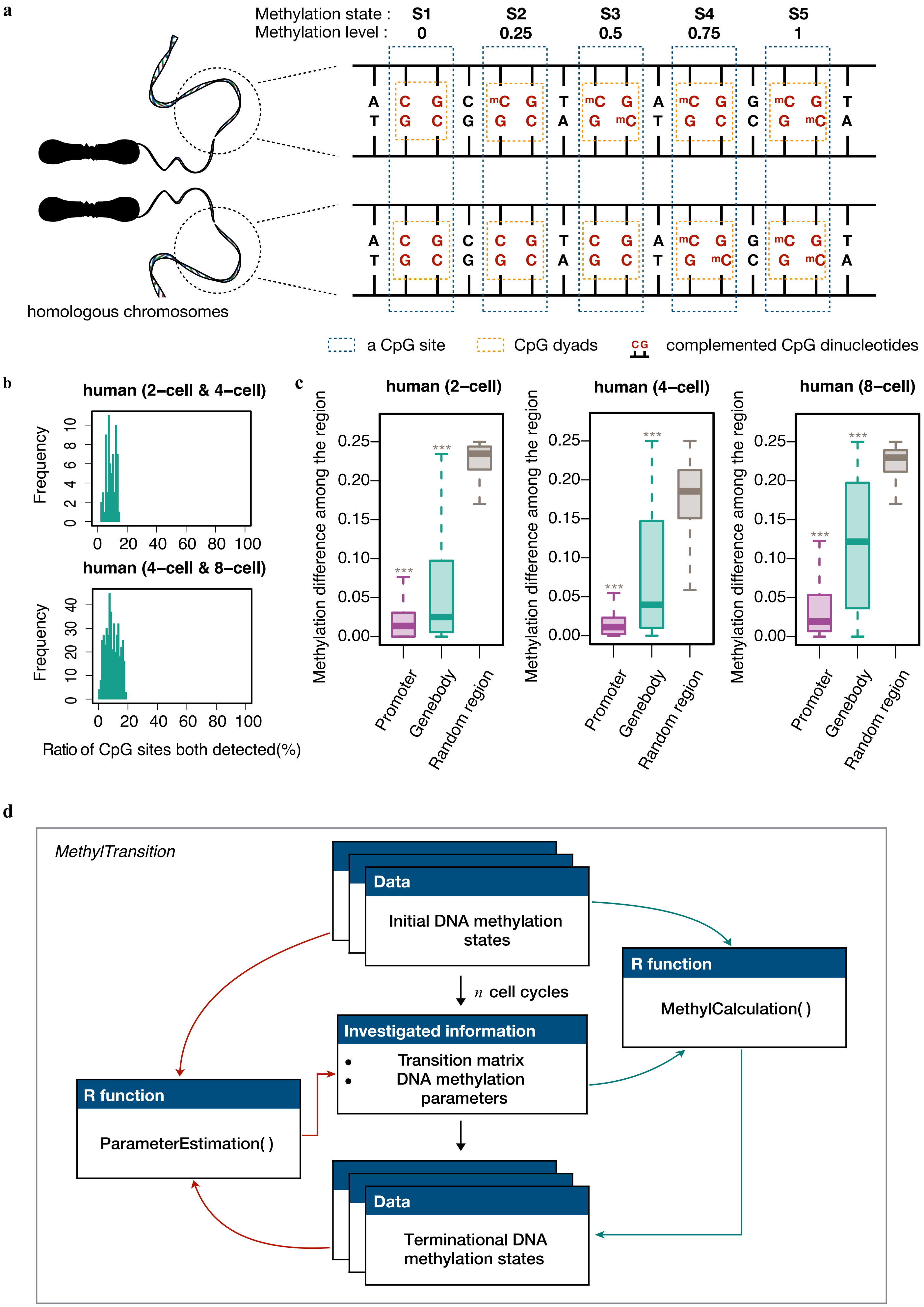

(legend on next page)

Fig. S1. Computational framework of MethylTransition

(a) Schematic diagram of CpG sites with different DNA methylation states. Here, a CpG site (blue box) contains two CpG dyads (orange boxes) in a diploid cell, while a CpG dyad contains two complemented CpG dinucleotides. (b) Histograms of the ratios of the both detected CpG sites between pairs of 2-cell stage and 4-cell stage embryos from the scBS-seq dataset (upper panel) and between pairs of 4-cell stage and 8-cell stage embryos from the scBS-seq dataset (lower panel). (c) Box plots of the methylation differences among promoters, gene bodies and random regions at the 2-cell, 4-cell and 8-cell stages during human embryogenesis. The methylation difference was measured as the variance in the DNA methylation level in a certain region. Student's t-test was performed for comparisons between promoters or gene bodies and random regions ("***": p-value < 0.001). (d) Schematic overview of the MethylTransition R library. MethylTransition has two main functions. The *ParameterEstimation* function can be used to estimate parameters with given initial and terminal DNA methylation states. The *MethylCalculation* function can be used to calculate the proportions of terminal DNA methylation states with given initial DNA methylation states and a set of parameters.

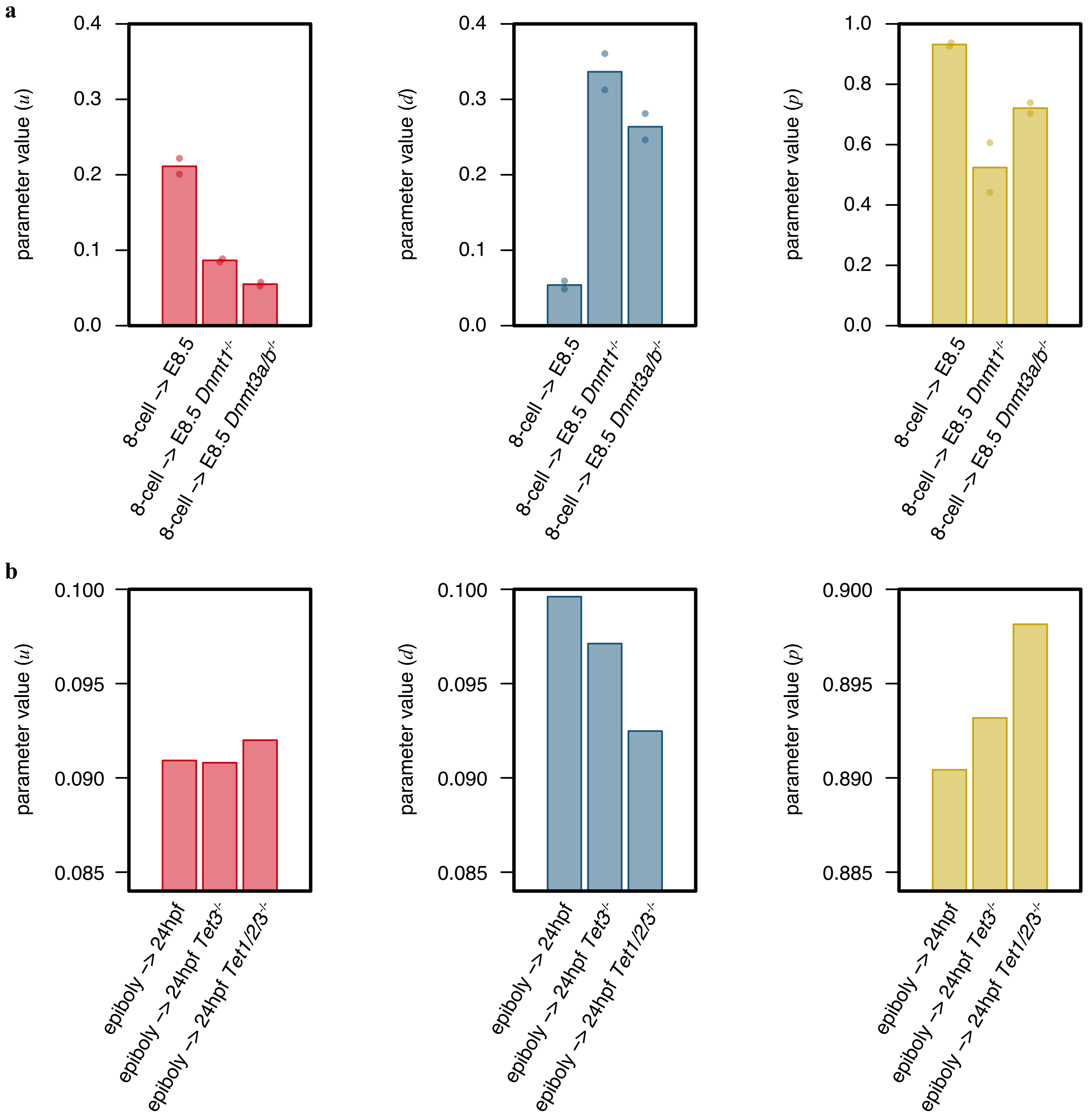

Fig. S2. Estimated parameters in DNA methylation enzyme knockout samples.

(a) Bar plot showing the parameter values estimated from the wild-type mouse embryos at the 8-cell stage to the wild-type and two types of enzyme-knockout mouse embryos (*Dnmt1*$^{-/-}$, *Dnmt3a/b*$^{-/-}$) at the E8.5 stage. Each dot represents the parameter values estimated using a biological replicate. (b) Bar plots showing the parameter values estimated from the wild-type zebrafish embryos at the epiboly stage to the wild-type and two types of enzyme-knockout zebrafish embryos (*Tet3*$^{-/-}$, *Tet1/2/3*$^{-/-}$) at the 24 hpf stage.

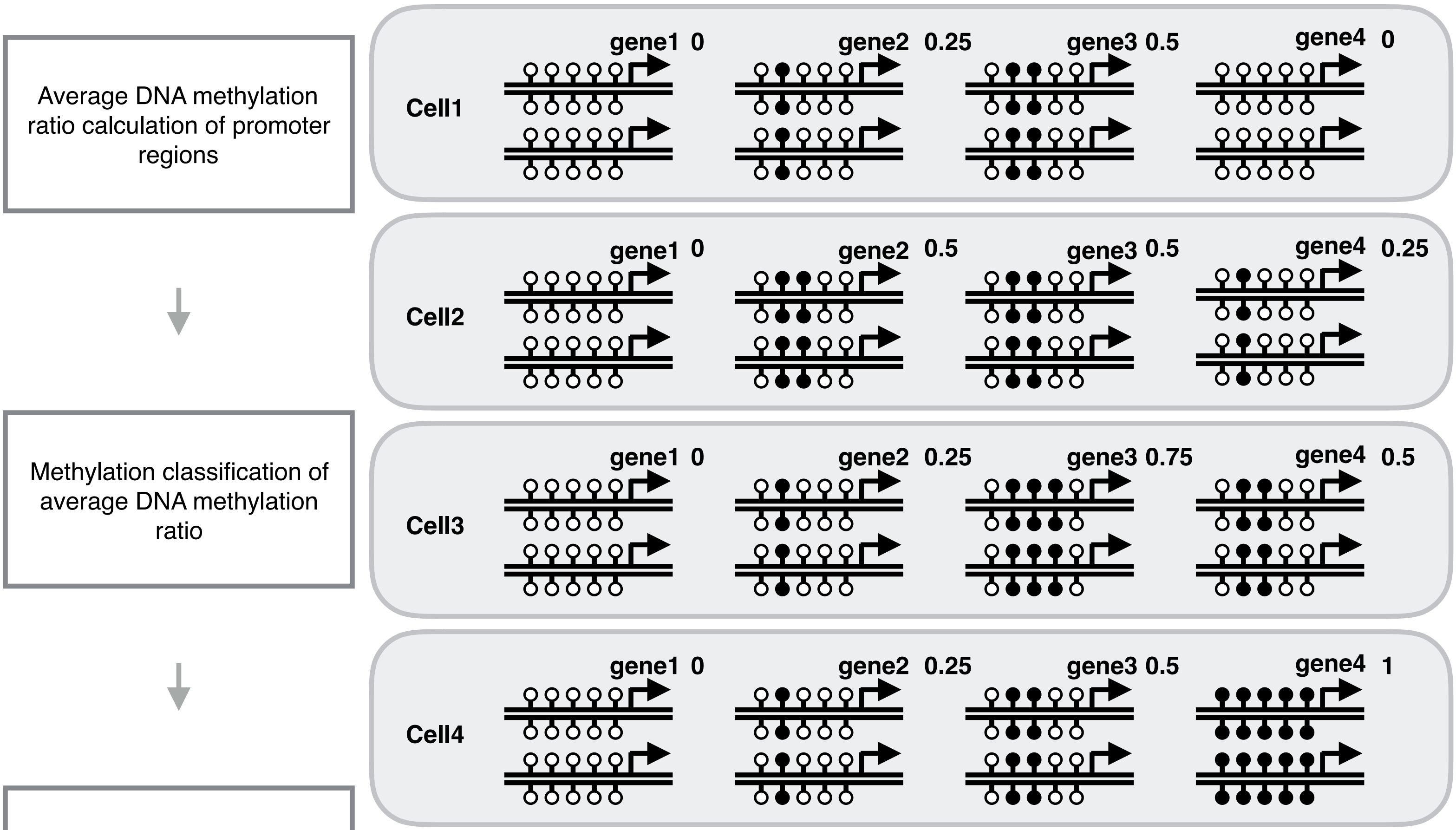

State	Proportion (%)
S1	100
S2	0
S3	0
S4	0
S5	0

State	Proportion (%)
S1	0
S2	75
S3	25
S4	0
S5	0

State	Proportion (%)
S1	0
S2	0
S3	75
S4	25
S5	0

State	Proportion (%)
S1	25
S2	25
S3	25
S4	0
S5	25

	gene1	gene2	gene3	gene4
Average methylation level	0	0.3125	0.5625	0.4375
Heterogeneity score	0.2	0.3	0.3	0.8

Fig. S3. Schematic workflow of the calculation of DNA methylation heterogeneity

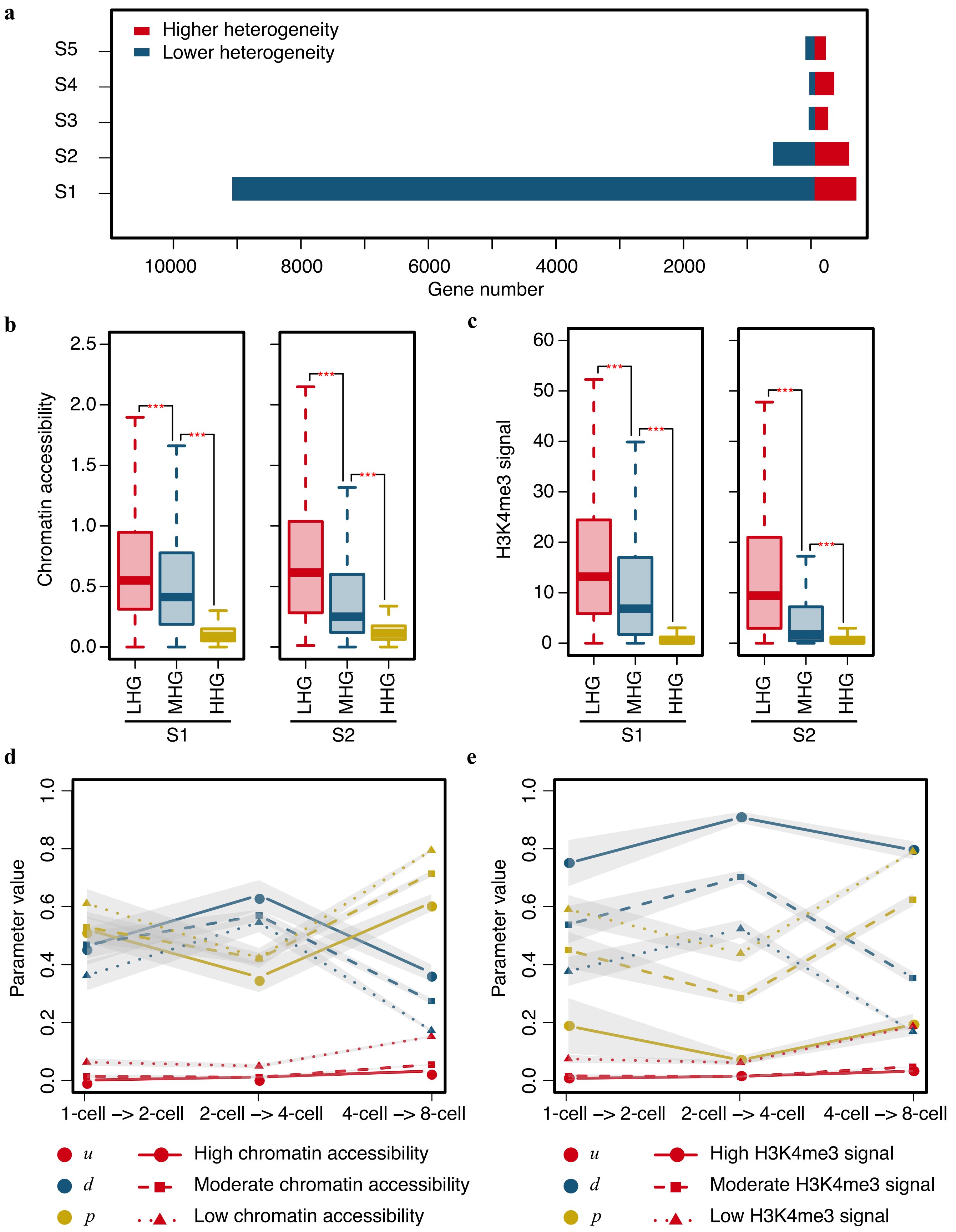

Fig. S4. Impacts of epigenetic features on DNA methylation heterogeneity

(a) Bar plot of the number of genes with higher predicted heterogeneity or lower predicted heterogeneity than observed heterogeneity in human 8-cell stage embryos.

(legend continued on next page)

(b) Box plots of the differences in chromatin accessibility between different categories of promoters in the S1 and S2 classes. The promoters in the S1 and S2 classes were divided into three categories: higher-heterogeneity gene promoters (HHGs; with observed heterogeneity higher than the 3rd quantile of the predicted heterogeneity score of the class), model-predictable heterogeneity gene promoters (MHGs) and lower-heterogeneity gene promoters (LHGs; with observed heterogeneity lower than the 1st quantile of the predicted heterogeneity score of the class). Student's t-test was performed for comparisons between adjacent categories ("***": p-value < 0.001). (c) Box plots of the differences in H3K4me3 signals between different categories of promoters in the S1 and S2 classes. (d) Estimated parameters for different groups of promoters with distinct chromatin accessibility during the first three cell cycles of human embryogenesis. The lines link the mean values of the parameters for each cell cycle. The gray shaded areas represent the 95% confidence intervals around the mean values. The promoters were grouped into three groups with high (≥ 0.8), moderate (between 0.2 and 0.8), or low (< 0.2) chromatin accessibility. Red indicates the parameter u, blue indicates the parameter d, and yellow indicates the parameter p. (e) Estimated parameters for different groups of promoters with distinct H3K4me3 signals during the first three cell cycles of human embryogenesis. The lines link the mean values of the parameters for each cell cycle. The gray shaded areas represent the 95% confidence intervals around the mean values. The promoters were grouped into three groups with high (≥ 15), moderate (between 3 and 15), or low (< 3) H3K4me3 signals. Red indicates the parameter u, blue indicates the parameter d, and yellow indicates the parameter p.

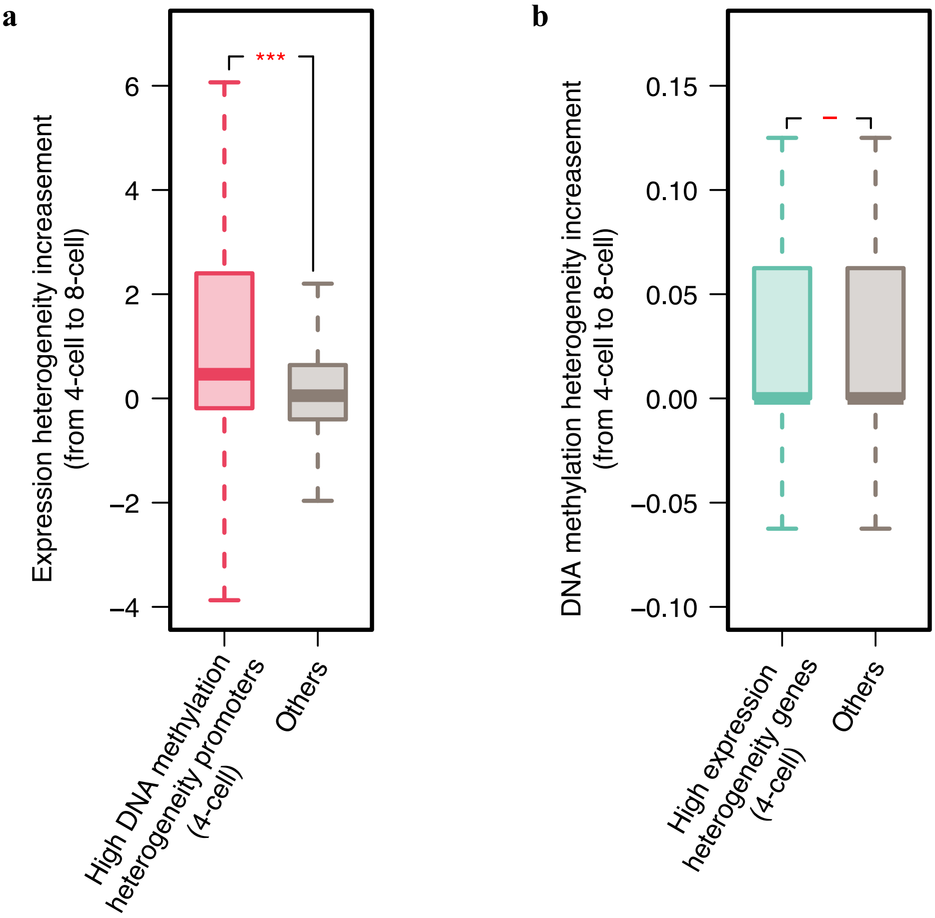

Fig. S5. The temporal association between promoter DNA methylation heterogeneity and expression heterogeneity. Box plots showing the expression heterogeneity increasement from the 4-cell stage to the 8-cell stage of the genes that have high DNA methylation heterogeneity promoters (DNA methylation heterogeneity > 0.3) at the 4-cell stage and other genes during human early embryogenesis. Student's t-test was performed for comparisons between these two classes ("***": p-value < 0.001). (b) Box plots showing the DNA methylation heterogeneity increasement from the 4-cell stage to the 8-cell stage of the genes that have high expression heterogeneity (expression heterogeneity > 0.5) at the 4-cell stage and others during human early embryogenesis. Student's t-test was performed for comparisons between these two classes ("-": p-value > 0.05).

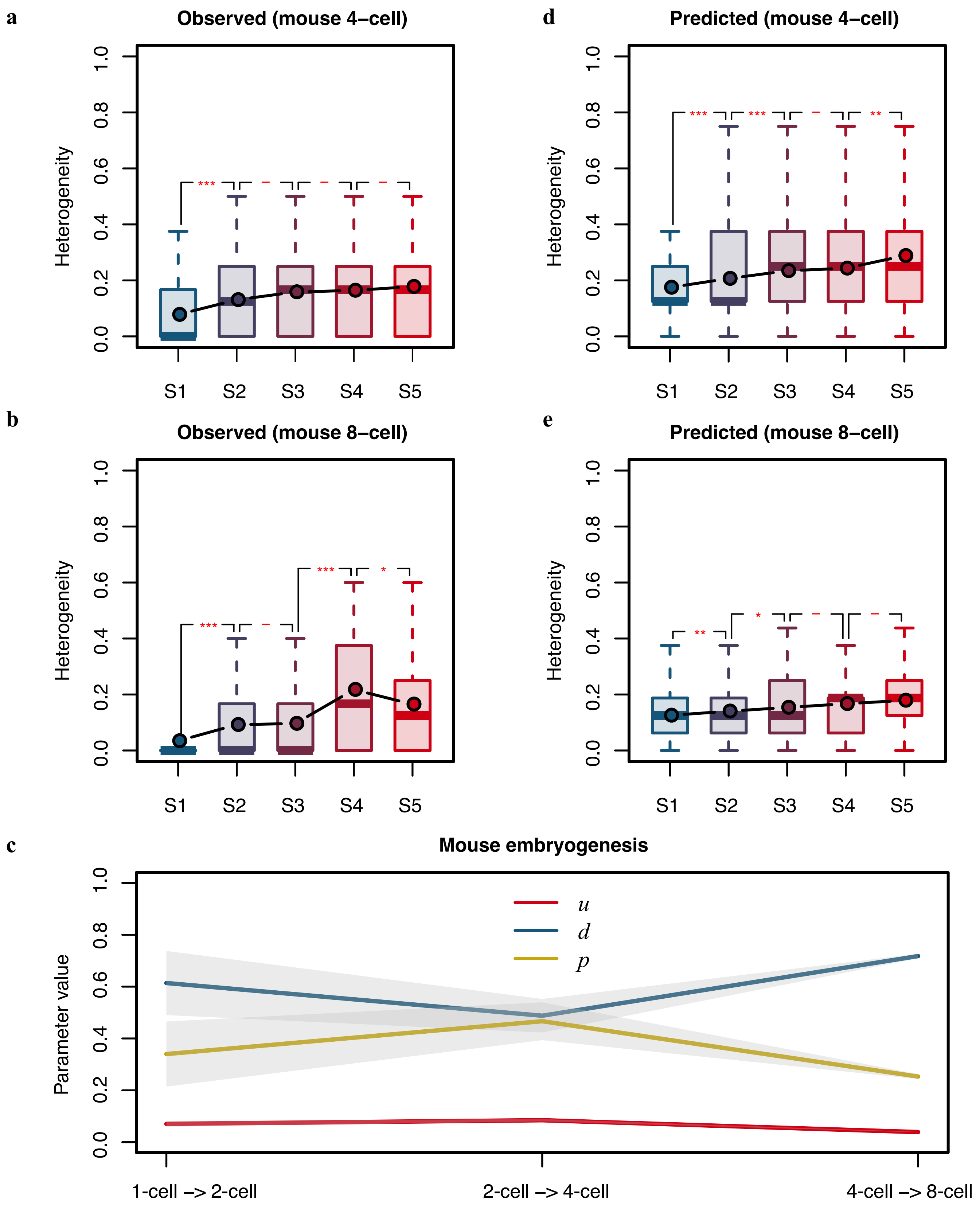

Fig. S6. Programmed DNA methylation heterogeneity in mouse pre-implantation embryos

(a, b) Box plots of the observed DNA methylation heterogeneity of promoters in the 4-cell stage (a) and 8-cell stage (b) during mouse embryogenesis. Promoters were classified into 5 classes based on their DNA

(legend continued on next page)

methylation states in the zygote stage. For each box, the point indicates the average DNA methylation heterogeneity of the class. Student's t-test was performed for comparisons between adjacent classes ("***": p-value < 0.001; "**": p-value < 0.01; "*": p-value < 0.05; "-": p-value > 0.05). The embryo with the largest number of high-quality scBS-seq data (more than 20% of all CpG sites detected) at each stage were used to calculate the DNA methylation heterogeneity. (c) Estimated parameters of the first three cell cycles during mouse embryogenesis. The lines link the mean values of the parameters for each cell cycle. The gray shaded areas represent the 95% confidence intervals around the mean values. (d, e) Box plots of the predicted DNA methylation heterogeneity of promoters in the 4-cell stage (d) and 8-cell stage (e) during mouse embryogenesis. Promoters were classified into 5 classes based on their DNA methylation states in the zygote stage. For each box, the point indicates the average DNA methylation heterogeneity of the class. Student's t-test was performed for comparisons between adjacent classes ("***": p-value < 0.001; "**": p-value < 0.01; "*": p-value < 0.05; "-": p-value > 0.05).
