# Supplementary material for: A DNA methylation state transition model reveals the programmed epigenetic heterogeneity in human pre-implantation embryos
Source: Genome Biol. 2020 Nov 16;21:277. doi: 10.1186/s13059-020-02189-8 (PMC7667739; doi:10.1186/s13059-020-02189-8)
Supplement: Supplementary file 3 — Additional file 3. Review history. [file 13059_2020_2189_MOESM3_ESM.docx]

Review History

**First round of review**

**Reviewer 1**

**Are you able to assess all statistics in the manuscript, including the appropriateness of statistical tests used?** Yes, and I have assessed the statistics in my report.

**Comments to author:**

In this article, Zhao et al. presented a probability model, MethylTransition, to characterize DNA methylation state transition during cell cycle. To overcome the missing value problem of single-cell bisulfite sequencing data, they first assign each promoter as one of the five discrete methylation states, i.e. 0%, 25%, 50%, 75% and 100% methylation. Then the authors came up with 3 parameters to depict the methylation state transition, i.e. the probabilities of de novo methylation, methylation maintenance and de-methylation. Applying Newton-Raphson algorithm, the optimal solution of the 3 parameters were defined by minimizing the difference between observed and predicted transition metrics. By simulating different levels of transition, they claimed the estimated parameters can represent the activity of methylation enzymes. In the second half of this paper, the authors presented a Gini-index based calculation of methylation heterogeneity. And they found that promoters with higher initial methylation tend to have higher heterogeneity in following cell cycle. The prediction of heterogeneity is improved if adjusting other confounding factors, like CpG ratio. Finally, they generated single-cell RNA-seq data and showed the correlation between methylation heterogeneity and gene expression variability. And the function of high heterogeneity genes are relevant to early embryogenesis. Overall MethylTransition would be a useful framework to characterize DNA methylation state transition during cell cycle, especially in the context of early development. There are several technical issues.

* As the authors mentioned, this model is based on two assumptions. "First, the DNA methylation states of CpG sites in the promoter of a gene are largely similar." I think this statement is somehow oversimplified. It's OK to use average methylation of all CpGs at a promoter as the feature, so you don't need to stick with this overstatement. "Second, the activity levels of DNA methylation-modifying enzymes in a cell are consistent across the genome." I think this one is lack of biological support, as we know that methylation can be impacted by chromatin activity. To solve such issue, I suggest to stratify promoters according to other features, like chromatin accessibility, CpG density, etc.

* For the parameter selection, the authors introduced de novo methylation, methylation maintenance and de-methylation. They are reasonable features of methylation regulation, but maybe not fully independent. Although it's difficult to fully separate them apart, it would be interesting to figure out how the 3 parameters are altered in methylation enzyme knockout samples.

* All results of this study are focused on promoter. Due to the missing value problem of single-cell sequencing data, I guess most of the promoters will have a methylation state of 0%, and some of them don't take methylation state change during cell cycle. So it raises my concern of the model robustness with such concentrated input. I suggest to use other genomic regions, like genebody and enhancer, to check whether the model still works well with more complex input. Furthermore, it would be convincing to remove the regions don't take state change before model training, and see whether the performance is still good.

**Reviewer 2**

**Are you able to assess all statistics in the manuscript, including the appropriateness of statistical tests used?** Yes, and I have assessed the statistics in my report.

**Comments to author:**

The paper by Zhao et al. presents an elegant mathematical model for dynamic changes of DNA methylation during cell division and applies the model to study early human/mouse embryo development. The model is based on a linear assumption of transition probabilities that involves three processes: active demethylation, maintenance, and de novo methylation, and the model parameters are inferred from single-cell BSseq data.  A highlight of the study is the ability to predict heterogeneous response of different gene promoters as a function of the initial DNA methylation level using few parameters. On the other hand, there are a number of weaknesses in this study. Perhaps due to its theoretical nature, the model assumptions, together with the values of the model parameters, may be difficult to verify directly. A number of mechanisms, such as DNMTs and TET family enzymes that are proposed to be dictating the properties of the dynamic model, but the roles of such enzymes are not quantitatively investigated or experimentally tested.  In terms of gene regulation, the negative correlation between promoter DNA methylation and gene expression levels has been well-established, and it is unclear what new insights have been brought by the current study.

Major comments:

- A strength of the model is its interpretability. The model parameters u, d, p are not abstract but can be related to specific biological processes (at least in principle). Therefore, it would be of high interest to compare with experimental data, such as kinetics and expression levels of DNMT and TET enzymes, to identifying factors that directly mediate these processes during embryonic development and assess their contribution by perturbation analyses.

- Single-cell BSseq data are used to infer model parameters, but it is unclear how such information is used exactly in the model. None of the equations involves cell-specific measurements.

- It is also unclear whether cell-to-cell variation at each development stage has been taken into account. Along the same line, the model assumes that DNA methylation level can be measured both before and after cell division. Since this obviously cannot be done in the experiment, an implicit assumption is that cells obtained from different embryos can be matched as if they are from the same cells. This assumption needs to be validated, for example, by using different cells from the same stage and testing the robustness of the model parameter values.

- Shouldn't the values of u, d, p be cell-specific? What is the range of these values among cells from the 2-cell stage? What is the implication of such variation in terms of cell-fate decision?  

- How conserved is the DNA methylation dynamics during mammalian early embryo development? There are public single-cell BSseq data in mouse, so it seems straightforward for the authors to apply their model. This would also justify their later experimental investigation using a mouse model.

Minor comments:

- the authors identified some correlation between variation of histone modification patterns and DNA methylation heterogeneity and interpreted it as causal relationship. This is probably an over-interpretation.  

- similarly, the relationship between DNA methylation and gene expression heterogeneity is also investigated at the level of correlation instead of causality. Perhaps this can be improved by analyzing the temporal differences between these modalities.

- How robust is the estimated values of u, d, p with respect to different criteria of promoter classification, subsampling, and cell-cell variation?  

- The procedure for predicting DNA methylation heterogeneity across different promoters is an essential part of the manuscript but not well described. Presumably this involves a random sampling model based on baseline DNA methylation level.  

- The role of Stella on DNA methylation is subtle. Some studies (ref. 32) suggest its role in maintenance of DNA methylation, but others (doi: 10.1038/s41421-019-0081-2) suggest it could also suppress hypermethylation. Some discussions of the possible alternative interpretations are encouraged.

**Authors Response**

**Point-by-point responses to the reviewers’ comments:**

*Reviewer #1:*

*In this article, Zhao et al. presented a probability model, MethylTransition, to characterize DNA methylation state transition during cell cycle. To overcome the missing value problem of single-cell bisulfite sequencing data, they first assign each promoter as one of the five discrete methylation states, i.e. 0%, 25%, 50%, 75% and 100% methylation. Then the authors came up with 3 parameters to depict the methylation state transition, i.e. the probabilities of de novo methylation, methylation maintenance and de-methylation. Applying Newton-Raphson algorithm, the optimal solution of the 3 parameters were defined by minimizing the difference between observed and predicted transition metrics. By simulating different levels of transition, they claimed the estimated parameters can represent the activity of methylation enzymes. In the second half of this paper, the authors presented a Gini-index based calculation of methylation heterogeneity. And they found that promoters with higher initial methylation tend to have higher heterogeneity in following cell cycle. The prediction of heterogeneity is improved if adjusting other confounding factors, like CpG ratio. Finally, they generated single-cell RNA-seq data and showed the correlation between methylation heterogeneity and gene expression variability. And the function of high heterogeneity genes are relevant to early embryogenesis. Overall MethylTransition would be a useful framework to characterize DNA methylation state transition during cell cycle, especially in the context of early development. There are several technical issues.*

** As the authors mentioned, this model is based on two assumptions. "First, the DNA methylation states of CpG sites in the promoter of a gene are largely similar." I think this statement is somehow oversimplified. It's OK to use average methylation of all CpGs at a promoter as the feature, so you don't need to stick with this overstatement. "Second, the activity levels of DNA methylation-modifying enzymes in a cell are consistent across the genome." I think this one is lack of biological support, as we know that methylation can be impacted by chromatin activity. To solve such issue, I suggest to stratify promoters according to other features, like chromatin accessibility, CpG density, etc.*

Response: We thank the reviewer for this helpful suggestion. In the revised manuscript, we modified our statements of the first assumption to "First, the DNA methylation states of CpG sites in the promoter of a gene can be represented by the average DNA methylation level in this promoter." and the second assumption to "Second, the activity levels of DNA methylation-modifying enzymes in genomic regions with similar chromatin status are largely consistent." in the revised manuscript. According to the reviewer’s suggestion, we also stratified the promoters into three groups (high, moderate, and low) based on their CpG ratios, chromatin accessibility, and H3K4me3 signal at zygote stage, respectively. Then, the transition parameters of the first three cell cycles of human embryogenesis on different groups of promoters were estimated using MethylTransition, respectively. These parameters showed a similar dynamic trend but distinct values (as shown in Figure 4d, Figure S4d, e in the revised manuscript), which is in agreement with the fact that the methylation can be impacted by chromatin activity.

** For the parameter selection, the authors introduced de novo methylation, methylation maintenance and de-methylation. They are reasonable features of methylation regulation, but maybe not fully independent. Although it's difficult to fully separate them apart, it would be interesting to figure out how the 3 parameters are altered in methylation enzyme knockout samples.*

Response: We agree with the reviewer that the changes of parameters in methylation enzyme knockout samples can help us to understand the biological meanings of the parameters. However, there is no single-cell BS-seq data during human or mouse early embryogenesis with methylation enzyme knockout. To overcome that difficulty, we extended the application of MethylTransition to bulk-cell level BS-seq data.

In the revised manuscript, we collected the BS-seq data in wild-type and two types of enzyme-knockout mouse embryos (*Dnmt1*^-/-^, *Dnmt3a/b*^-/-^) at the E8.5 stage (GSE130735). As shown in Figure S2a, *Dnmt1*^-/-^ embryos indeed displayed the smallest *p* (the probability of methylation maintenance), and *Dnmt3a/b*^-/-^ embryos displayed the smallest *u* (the probability of *de novo* methylation). In addition, we also collected the BS-seq data in wild-type and two types of enzyme-knockout zebrafish embryos (*Tet3*^-/-^, *Tet1/2/3*^-/-^) at 24 hpf (GSE68087). As shown in Figure S2b, both *Tet3*^-/-^ embryos and *Tet1/2/3*^-/-^ embryos showed smaller *d* (the probability of active demethylation) than wild-type embryos, and *Tet1/2/3*^-/-^ embryos showed the smallest *d*.

Our results indicate that those parameters are largely interpretable. Nevertheless, we fully agree with the reviewer that the DNA methylation regulation features are not fully independent, and we added the discussion of the complex relationship between parameters and enzyme activities in the revised manuscript.

** All results of this study are focused on promoter. Due to the missing value problem of single-cell sequencing data, I guess most of the promoters will have a methylation state of 0%, and some of them don't take methylation state change during cell cycle. So it raises my concern of the model robustness with such concentrated input. I suggest to use other genomic regions, like genebody and enhancer, to check whether the model still works well with more complex input. Furthermore, it would be convincing to remove the regions don't take state change before model training, and see whether the performance is still good.*

Response: It should be mentioned that, in our study, the methylation states of some promoters are assigned as NA (not available) due to the missing values at those loci. The NA state is different from un-methylated state. To avoid the missing value problem of single-cell BS-seq data, the promoters with states as NA are removed from parameter estimation. We added the description of NA state in the revised Methods section.

Based on the reviewer’s suggestion, we applied MethylTransition on genebody and enhancer, respectively. For each genebody or enhancer, we calculated its average methylation level to represent its methylation state. Here, enhancers are defined as ATAC-seq peaks detected at the 2-cell, 4-cell, or 8-cell stages of human embryos (GSE101571), which are located at the distal upstream (2k~50k) of TSS. As shown in Figure R1, the estimated parameters during the first three cell cycles based on genebody or enhancers indeed showed similar dynamic trends to those estimated based on promoters (Figure 3c), confirming the robustness of MethylTransition model.

**Figure R1.** Estimated parameters based on the genebody (a) and enhancers (b) of the first three cell cycles during human embryogenesis.

*Reviewer #2:*

*The paper by Zhao et al. presents an elegant mathematical model for dynamic changes of DNA methylation during cell division and applies the model to study early human/mouse embryo development. The model is based on a linear assumption of transition probabilities that involves three processes: active demethylation, maintenance, and de novo methylation, and the model parameters are inferred from single-cell BSseq data.  A highlight of the study is the ability to predict heterogeneous response of different gene promoters as a function of the initial DNA methylation level using few parameters. On the other hand, there are a number of weaknesses in this study. Perhaps due to its theoretical nature, the model assumptions, together with the values of the model parameters, may be difficult to verify directly. A number of mechanisms, such as DNMTs and TET family enzymes that are proposed to be dictating the properties of the dynamic model, but the roles of such enzymes are not quantitatively investigated or experimentally tested.  In terms of gene regulation,* *the negative correlation between promoter DNA methylation and gene expression levels has been well-established, and it is unclear what new insights have been brought by the current study.*

*Major comments:*

*- A strength of the model is its interpretability. The model parameters u, d, p are not abstract but can be related to specific biological processes (at least in principle). Therefore, it would be of high interest to compare with experimental data, such as* *kinetics and expression levels of DNMT and TET enzymes, to identifying factors that directly mediate these processes during embryonic development and assess their contribution by perturbation analyses.*

Response: We thank the reviewer for this helpful suggestion. Based on the reviewer’s suggestion, we examined the expression levels of DNA methylation enzymes through literature searching. Petrussa *et al.* reported that DNMT1 protein level in nuclear increases from 2-cell to 8-cell stage in human embryos (PMID: 24994815), which is in agreement with our results that *p* (the probability of methylation maintenance) increases from “2-cell to 4-cell” transition to “4-cell to 8-cell” transition (Figure 3c). Different from human, Gao *et al*. reported that DNMT1 showed the highest protein level at 4-cell stage in mouse embryos (PMID: 29281840), which is supported by our results that *p* showed the highest value at “2-cell to 4-cell” transition (Figure S6c). Petrussa *et al.* also reported that DNMT3a and DNMT3b are largely absent during the first three cell cycles of human embryogenesis, which is in agreement with the small values of *u* (the probability of *de novo* methylation) estimated for the three cell cycles (Figure 3c). Guo *et al* reported the expression level of *TET3* decreases from zygote to 8-cell stage of human embryos, while the expression level of *TET2* transiently increases at 4-cell stage (PMID: 25079557). Due to the different dynamic trends of *TET2* and *TET3*, it is difficult to link the expression level of TET families to the overall demethylation activity which is presented as parameter *d*.

We further investigated the changes of estimated parameters in methylation enzyme knockout samples. As described in the response to Reviewer #1, the parameters of our model are largely interpretable (Figure S2). Nevertheless, we agree that the DNA methylation regulation features are not fully independent, and we added the discussion of the complex relationship between parameters and enzyme activities in the revised manuscript.

*- Single-cell BSseq data are used to infer model parameters, but it is unclear how such information is used exactly in the model. None of the equations involves cell-specific measurements.*

Response: Single-cell BS-seq data are used to infer the ratio of each DNA methylation state within a single cell, described in formula 7 and 9 in the revised manuscript. Such information was used to calculate the state transition frequency matrix (i.e. $T_{B}$ in the formula 9), which is the key step for parameter estimation. We added detailed descriptions in the Methods section of the revised manuscript.

*- It is also unclear whether cell-to-cell variation at each development stage has been taken into account. Along the same line, the model assumes that DNA methylation level can be measured both before and after cell division. Since this obviously cannot be done in the experiment, an implicit assumption is that cells obtained from different embryos can be matched as if they are from the same cells. This assumption needs to be validated, for example, by using different cells from the same stage and testing the robustness of the model parameter values.*

Response: We agree with the reviewer that the robustness of our model using different cells should be evaluated. In the revised manuscript, we tested the robustness of our model parameter values using different cells’ BS-seq data from the same stage. As shown in updated Figure 2a, parameters estimated by using different cells from the same stage are quite robust.

*- Shouldn't the values of u, d, p be cell-specific? What is the range of these values among cells from the 2-cell stage? What is the implication of such variation in terms of cell-fate decision?*

Response: As we mentioned above, the parameter values of *u*, *d*, *p* are quite robust by using different cells’ BS-seq data from the same stage (shown in Figure 2a). For “2-cell to 4-cell transition”, the range of *u* is 0.050 (min: 0.005, max: 0.055), *d* is 0.244 (min: 0.416, max: 0.660) and *p* is 0.246 (min: 0.331, max: 0.577). Whether such a variation can play a role on the cell-fate decision is an interesting topic. However, our results did not provide a direct evidence for this topic, which is worth future investigation.

*- How conserved is the DNA methylation dynamics during mammalian early embryo development? There are public single-cell BSseq data in mouse, so it seems straightforward for the authors to apply their model. This would also justify their later experimental investigation using a mouse model.*

Response: We applied MethylTransition to mouse pre-implantation embryos using the single-cell DNA methylome data (GSE78140). The parameter values of *u*, *d* and *p* for the first three cell cycles of mouse embryogenesis were shown in Figure S6c. Similar to that in human embryos, the values of *u* (the probability of *de novo* methylation) are very small for the first three cell cycles of mouse embryos. For the values of *p* (the probability of methylation maintenance) and *d* (the probability of active demethylation), their dynamic trends have differences between human and mouse embryos. During mouse early embryogenesis, *p* displayed the largest value in the “2-cell to 4-cell” transition, and *d* displayed the smallest value in that transition. In contract, during human early embryogenesis, *p* displayed the smallest value in the “2-cell to 4-cell” transition, and *d* displayed the largest value in that transition. Such a difference is supported by the observations that 4-cell stage showed higher DNA methylation level than 2-cell stage and 8-cell stage in mouse embryos (PMID: 28621329), while 4-cell stage showed lower DNA methylation level than 2-cell stage and 8-cell stage in human embryos (PMID: 25079557). The results demonstrated that the parameters of our model can capture the difference of DNA methylation dynamics between species.

*Minor comments:*

*- the authors identified some correlation between variation of histone modification patterns and DNA methylation heterogeneity and interpreted it as causal relationship. This is probably an over-interpretation.*

Response: We have toned down the statement in the revised manuscript.

*- similarly, the relationship between DNA methylation and gene expression heterogeneity is also investigated at the level of correlation instead of* *causality. Perhaps this can be improved by analyzing the temporal differences between these modalities.*

Response: We thank the reviewer for this suggestion. To analyze the temporal differences between the DNA methylation heterogeneity and expression heterogeneity, we investigated the association between one type of heterogeneity at the 4-cell stage and the change of another type of heterogeneity at the 8-cell stage. As shown in updated Figure S5a, the genes with high DNA methylation heterogeneity promoters at the 4-cell stage showed larger expression heterogeneity increasement from that stage to the 8-cell stage than other genes. Differently, the genes with high expression heterogeneity at the 4-cell stage showed similar promoter DNA methylation heterogeneity increasement from that stage to the 8-cell stage with other genes. The result implied that DNA methylation heterogeneity at promoters might affect the heterogeneity of gene expression. We added the above analysis in the revised manuscript.

*- How robust is the estimated values of u, d, p with respect to different criteria of promoter classification, subsampling, and cell-cell variation?*

Response: We investigated the robustness of estimated parameters upon subsampling (i.e. the effect of dropout), and as shown in Figure 2b, the estimated values were robust even with a high dropout rate. In the revised manuscript, we also tested the robustness of our model parameter values using different cells’ BS-seq data from the same stage. As shown in updated Figure 2a, parameters estimated by using different cells from the same stage are quite robust. In terms of promoter classification, we stratified the promoters into three groups (high, moderate, and low) based on their CpG ratios, chromatin accessibility, and H3K4me3 signal at zygote stage, respectively. Then, the transition parameters of the first three cell cycles of human embryogenesis on different groups of promoters were estimated, respectively. These parameters showed a similar dynamic trend but distinct values (as shown in Figure 4d, Figure S4d, e in the revised manuscript), which is in agreement with the fact that the methylation can be impacted by chromatin activity.

*- The procedure for predicting DNA methylation heterogeneity across different promoters is an essential part of the manuscript but not well described. Presumably this involves a random sampling model based on baseline DNA methylation level.*

Response: We added a detailed description of the procedure for predicting DNA methylation heterogeneity in the revised manuscript. A random sampling method (based on R function *sample()*) was used to assign each promoter a state in each of the daughter cells based on the calculated probability and the initial DNA methylation state in the mother cell. For different cells in the same stage, different random seeds were used.

*- The role of Stella on DNA methylation is subtle. Some studies (ref. 32) suggest its role in maintenance of DNA methylation, but others (doi: 10.1038/s41421-019-0081-2) suggest it could also suppress hypermethylation. Some discussions of the possible alternative interpretations are encouraged.*

Response: We added the discussion of the possible alternative interpretation of Stella in the revised manuscript.

**Second round of review**

**Reviewer 1**

The authors have addressed all my concerns.

**Reviewer 2**

I applaud the authors for an outstanding revision. The new analyses have satisfactorily addressed all my concerns.
